# Symptoms and Conditions in Children and Adults up to 90 Days after SARS-CoV-2 Infection: A Retrospective Observational Study Utilizing the Common Data Model

**DOI:** 10.3390/jcm13102911

**Published:** 2024-05-15

**Authors:** Minjung Han, Taehee Chang, Hae-ryoung Chun, Suyoung Jo, Yeongchang Jo, Dong Han Yu, Sooyoung Yoo, Sung-il Cho

**Affiliations:** 1 Graduate School of Public Health, Seoul National University, Seoul 08826, Republic of Korea; mhanmhan@snu.ac.kr (M.H.); redwood0226@snu.ac.kr (T.C.); mamimi777@snu.ac.kr (H.-r.C.); 2 Chaum Life Center, CHA University School of Medicine, Seoul 06062, Republic of Korea; 3 Institute of Health and Environment, Seoul National University, Seoul 08826, Republic of Korea; josuyoung1@snu.ac.kr; 4 Department of Preventive Medicine, Yonsei University College of Medicine, Seoul 03722, Republic of Korea; platypus825@gmail.com; 5 Big Data Department, Health Insurance Review and Assessment Service, Wonju 26465, Republic of Korea; donghan86@hira.or.kr; 6 Healthcare ICT Research Center, Seoul National University Bundang Hospital, Seongnam 13620, Republic of Korea; yoosoo0@gmail.com

**Keywords:** persistent symptoms of COVID-19, long COVID, post-COVID condition, post-acute sequelae of COVID-19

## Abstract

**Background/Objectives:** There have been widespread reports of persistent symptoms in both children and adults after SARS-CoV-2 infection, giving rise to debates on whether it should be regarded as a separate clinical entity from other postviral syndromes. This study aimed to characterize the clinical presentation of post-acute symptoms and conditions in the Korean pediatric and adult populations. **Methods**: A retrospective analysis was performed using a national, population-based database, which was encoded using the Observational Medical Outcomes Partnership (OMOP) Common Data Model (CDM). We compared individuals diagnosed with SARS-CoV-2 to those diagnosed with influenza, focusing on the risk of developing prespecified symptoms and conditions commonly associated with the post-acute sequelae of COVID-19. **Results**: Propensity score matching yielded 1,656 adult and 343 pediatric SARS-CoV-2 and influenza pairs. Ninety days after diagnosis, no symptoms were found to have elevated risk in either adults or children when compared with influenza controls. Conversely, at 1 day after diagnosis, adults with SARS-CoV-2 exhibited a significantly higher risk of developing abnormal liver function tests, cardiorespiratory symptoms, constipation, cough, thrombophlebitis/thromboembolism, and pneumonia. In contrast, children diagnosed with SARS-CoV-2 did not show an increased risk for any symptoms during either acute or post-acute phases. **Conclusions**: In the acute phase after infection, SARS-CoV-2 is associated with an elevated risk of certain symptoms in adults. The risk of developing post-acute COVID-19 sequelae is not significantly different from that of having postviral symptoms in children in both the acute and post-acute phases, and in adults in the post-acute phase. These observations warrant further validation through studies, including the severity of initial illness, vaccination status, and variant types.

## 1. Introduction

The COVID-19 pandemic has unleashed a global health crisis of unparalleled scale. Originating from the RNA-based SARS-CoV-2 virus, the first case of COVID-19 was identified in Wuhan, China, in December 2019 [1]. As of now, the disease has led to over 774 million cases and 7 million fatalities worldwide [2]. Beyond acute infections, SARS-CoV-2 has been associated with protracted symptoms persisting six months or longer in certain patients. These symptoms, which range from dyspnea and cough to fatigue and cognitive deficits, significantly disrupt daily activities [3]. Such post-infection manifestations are referred to by various terms, including post-acute sequelae of SARS-CoV-2 infection (PASC), long COVID, and post-COVID conditions (PCC). The World Health Organization (WHO) characterizes PASC as a spectrum of symptoms that persist or emerge three months after diagnosis, last for at least two months, and cannot be attributed to an alternative diagnosis. Meta-analyses have shown that 43–80% of adults who contract the virus report at least one persistent symptom [4,5,6].

The symptomatic profile of PASC bears similarities to postviral syndromes resulting from other viral infections, such as those caused by influenza [7]. The hallmark features of these syndromes include exercise intolerance, profound fatigue, cognitive disturbances, myalgia, and arthralgia, among other nonspecific symptoms [7]. The clinical overlap suggests that a potential shared pathophysiological mechanism exists between PASC and other postviral syndromes. Theories on the pathogenesis of PASC include persistent viral component activation leading to chronic inflammation, autoimmune responses via autoantibodies, and direct tissue damage [8,9,10]. Similar mechanisms have been suggested for postviral syndrome, including viral persistence, autoimmunity, and alterations in the microbiome [7]. Despite these parallels, it is not yet clear whether PASC is simply an extension of known postviral syndromes. Understanding whether PASC is a unique entity, distinct from other postviral conditions such as those following influenza, is crucial for developing appropriate clinical interventions.

Additionally, the differences between the clinical presentations of PASC between children and adults warrant further investigation. While adult patients report post-acute symptoms at a rate of 10–61%, children exhibit such symptoms at a significantly lower rate of 1–30% [11]. Studies indicate a decline in the prevalence of PASC over time, with adult rates falling from 50% to 34% and children’s rates from 20% to 11% over a period of 6 to 12 months [12]. Common persistent symptoms in adults include fatigue and memory issues [6,13], while in children, fatigue, respiratory difficulties, exercise intolerance, weakness, and challenges with mobility are frequently observed [14]. Evidence suggests that there may be differences in the prevalence, duration, and intensity of PASC between adults and children. Nevertheless, these distinctions have not been specifically studied within the Korean population. Also, the causal pathways driving these differences remain to be elucidated. 

To address this gap, our study examines the incidence and risk of post-acute symptoms and conditions following SARS-CoV-2 infection in Korean children and young people (CYP), as well as in adults. Utilizing a national database transformed into the Common Data Model (CDM), which standardizes medical data for interorganizational research, we investigate the sequelae of COVID-19 at 1, 30, and 90 day intervals at the population level. This study aims to describe the symptomatology of post-acute SARS-CoV-2 infection in the Korean population. By using influenza as a control, we aim to evaluate whether the postviral symptoms of SARS-CoV-2 are notably different from those associated with other viral infections. 

## 2. Materials and Methods

### 2.1. Study Design and Data Source

This was a population-based, retrospective, observational comparative cohort study. The data source for this study was the Health Insurance Review and Assessment Service COVID-19 Observational Medical Outcomes Partnership (HIRA COVID-19 OMOP) database [15]. The Health Insurance Review and Assessment Service (HIRA) is in charge of claims reviews and maintaining a data warehouse of the National Health Insurance (NHI) of South Korea. To facilitate COVID-19 research during the pandemic, HIRA developed an infrastructure to receive analytic codes from external researchers and apply them to a database consisting of 20% of the population who were eligible for NHI in 2021 (approximately 10 million people). This sample was extracted using a randomized sampling method based on sex and age to ensure representativeness. The claims data of this sample of patients from 1 January 2018 to 30 April 2022 were transformed into the standard vocabulary concepts and schema of the OMOP Common Data Model (CDM) version 5.3.1., which ensures uniformity in the semantics and structure of healthcare data and facilitates analysis without the disclosure of personal information. [16,17]. The claims data included information on demographics (age, sex, insurance type), medical history (diagnostic codes, procedures, treatment), drug prescription history, visit type and mortality. COVID-19 diagnoses based on reverse transcription–polymerase chain reaction (RT–PCR) from the Korea Disease Control and Prevention Agency were linked to claims data to ensure the validity of COVID-19 confirmed cases. 

The Observational Medical Outcome Partnership (OMOP) Common Data Model (CDM) is maintained by the Observational Health Data Sciences and Informatics (OHDSI) network and is utilized throughout the world to facilitate distributed network research without the disclosure of patient-level data [18]. A detailed description of the HIRA COVID OMOP database, data collection process and methodology can be found elsewhere [15]. 

### 2.2. Study Participants

The study population consisted of pediatric and adult patients. The pediatric target cohort consisted of those aged less than or equal to 19 years with a diagnosis of SARS-CoV-2 between 1 January 2021 and 30 September 2021 for the first time in the person’s history. The adult target cohort was defined as those greater than 19 years with a diagnosis of SARS-CoV-2 between 1 January 2021 and 30 September 2021 for the first time in the person’s history. Those who did not have a continuous observation period of at least 90 days prior to the date of diagnosis were excluded from both cohorts. SARS-CoV-2 was defined as the occurrence of conditions and observations as defined in Supplementary Table S8.

The control cohorts for children and adults were defined as follows: the pediatric control cohort was defined as those less than or equal to 19 years with a diagnosis of influenza between 1 January 2021 and 30 September 2021 for the first time in the person’s history. The adult control cohort was defined as those greater than 19 years with a diagnosis of influenza between 1 January 2021 and 30 September 2021 for the first time in the person’s history. Those without a continuous observation period of at least 90 days prior to the date of diagnosis were excluded. Influenza was defined as the occurrence of conditions and observations as defined in Supplementary Table S9.

### 2.3. Outcomes

The primary outcome was defined as the occurrence of symptoms that were reported in the literature to be associated with PASC [19,20]. A previous report identified 121 syndromic/systemic clusters of ICD-10 codes that were reviewed by medical experts and clinically predicted to be associated with the post-acute sequelae of COVID-19 [21]. Another report aggregated 96 health outcomes into 13 diagnosis/symptom complexes and 3 outcome domains (physical health, mental health, and physical/mental overlap) based on the previous literature and clinical expertise [20]. Based on these reports, we selected 57 symptoms/syndromes that were reported to be associated with SARS-CoV-2. The concept IDs corresponding to these symptoms and conditions can be found in Supplementary Table S10.

Outcome assessment follow-up time, or risk window, spanned 1, 30, and 90 days from the index date to 180 days from the index date, or up to 30 April 2022, whichever event came first [21]. Those who died due to any cause were censored. Only the outcomes without missing values for relative risk, 95% confidence interval, and *p*-value were included in the final analysis.

### 2.4. Statistical Analyses

Statistical analysis was performed based on the OHDSI’s ATLAS tool version 2.7.6 (https://www.ohdsi.org/web/atlas, accessed on 9 April 2024). ATLAS, an interactive analysis platform, was developed by the OHDSI network as a tool to facilitate the transformation and analysis of data [18]. An analytic R package generated by ATLAS was sent to HIRA where it was executed on the HIRA COVID-19 OMOP database. Only the output containing summarized results excluding patient-level data was returned. Further details on the analytic method can be found elsewhere [15]. 

The incidence proportion of an outcome per 1000 persons was calculated by dividing the number of incident cases by the total person count and multiplying this value by 1000. The incidence rate of an outcome per 1000 person-years was calculated by dividing the number of incident cases by the time-at-risk (person-years) and multiplying the results by 1000. To control for differences in baseline characteristics between the target (SARS-CoV-2) and control (influenza) cohorts, we performed 1: maximum propensity score (PS)-adjusted matching. PS was estimated with regularized logistic regression using the following covariates: age groups (in intervals of five years), gender, index month, index year, and Charlson comorbidity index (CCI). The width of the caliper was 0.5 standardized logits. Covariate balance metrics were used to evaluate the success of measured confounding control, defined as all covariates having a standardized mean difference (SMD) less than 0.1 (Figure S4). We utilized the distribution of preference scores (defined as a transformation of PS adjusted to account for variations in prevalence between populations) to assess equipoise, which is characterized by the majority of subjects having propensity score-based preference scores ranging from 0.25 to 0.75 [17,22] (Figures S5 and S6). We then calculated the hazard ratios (HR) of symptoms and conditions and 95% confidence interval (CI) using a Cox proportional hazards model. We used Kaplan–Meier survival plots to assess the assumption of proportionality (Figures S7 and S8). The analysis was performed using R version 4.0.5 (The R Foundation for Statistical Computing, Vienna, Austria). Statistical significance was defined by a *p*-value of less than 0.05. 

Only the first exposures per subject were included. For those with both influenza and SARS-CoV-2 diagnoses, only the first diagnosis was counted. Subjects with the outcome prior to the risk window start were excluded from the analysis. Sensitivity analyses were performed using different ratios for PS matching and regression models to assess the robustness of results. In addition to 1: maximum PS matching, 1:1 PS matching was performed. Also, in addition to Cox regression, Poisson regression was used in the sensitivity analyses to calculate relative risk.

### 2.5. Ethical Statement

The study was approved by the Institutional Review Board (IRB) of Seoul National University (approval number: E2308/001-018) and was allowed to waive the requirement for informed consent.

## 3. Results

### 3.1. Baseline Characteristics

The total number of patients in the HIRA database (reference date: 1 July 2022) was 9,822,577 (≤19 years: 2,577,969 individuals, >19 years: 7,244,608 individuals). After excluding those who did not fit the inclusion criteria and performing 1: maximum PS matching, the final study population included 343 and 1656 target–comparator pairs for children and adults, respectively (Figures S2 and S3). The follow-up time was similar for the adult SARS-COV-2 and influenza cohorts, at a target and comparator median follow-up time of 91 days. The follow-up time was also similar for the pediatric SARS-COV-2 and influenza cohorts, with a median follow-up time of 91 days for both groups. 

In the pediatric study population, the influenza cohort tended to be younger than the SARS-CoV-2 cohort before PS matching (Table S1). There was a greater proportion of children 1–4 years of age in the influenza cohort (SARS-CoV-2, 1–4 years: 16.3%; influenza, 1–4 years: 42.0%) (Table S1). In terms of gender, both the influenza cohort and the SARS-COV-2 cohort prior to PS matching had relatively even proportions among groups (SARS-CoV-2, female: 45.5%; influenza, female: 51.9%) (Table S1). In the adult study population, there was a greater proportion of younger individuals in the SARS-CoV-2 cohort (SARS-CoV-2, 19–39 years: 57.4%; influenza, 19–39 years: 26.3%) (Table S2). In terms of gender, participants were evenly distributed between the SARS-CoV-2 and influenza cohorts (SARS-CoV-2, female: 48.2%; influenza, female: 54.2%) (Table S2). 

Baseline characteristics were balanced for the PS-matched cohorts, with standardized mean differences (SMD) < 0.2 for most of the covariates (Figure S4). This suggests that the cohorts were well-balanced in terms of baseline characteristics after propensity score matching. The distribution of preference scores for children and adults is shown in Figures S5 and S6. Since the majority of patients in each comparison pair had preference scores between 0.25 and 0.75, we concluded that there was sufficient empirical equipoise and that the target–comparator pairs were comparable (Figures S5 and S6) [22,23].

### 3.2. Incidence Rates of Symptoms and Conditions at 90 Days after the Diagnosis Date

The incidence rates of symptoms and conditions per 1000 person-years at 90 days after the diagnoses date tended to be lower in the SARS-CoV-2 cohort for both children and adults (Tables S3 and S4). The outcomes that showed higher incidence rates in the SARS-CoV-2 cohort for children included abnormal liver function test (IR SARS-CoV-2: 9.27, 95% CI: 7.90–10.64; IR influenza: 7.91, 95% CI: 2.32–13.49), arthralgia (IR SARS-CoV-2: 37.35, 95% CI: 34.45–40.24; IR influenza: 25.53, 95% CI: 15.11–35.96), dizziness/syncope (IR SARS-CoV-2: 20.10, 95% CI: 18.05–22.16; IR influenza: 16.26, 95% CI: 8.13–24.39) (Table S3). The outcomes that showed higher incidence rates in the SARS-CoV-2 cohort for adults included hair loss (IR SARS-CoV-2: 6.00, 95% CI: 5.55–6.44; IR influenza: 4.31, 95% CI: 2.38–6.24), anxiety symptoms (IR SARS-CoV-2: 1.91, 95% CI: 1.66–2.16; IR influenza: 1.73, 95% CI: 0.51–2.95), myalgia (IR SARS-CoV-2: 104.59, 95% CI: 101.89–107.30; IR influenza: 100.00, 95% CI: 84.38–115.62), ocular symptoms (IR SARS-CoV-2: 99.71, 95% CI: 97.22–102.20; IR influenza: 95.33, 95% CI: 81.43–109.24), myositis (IR SARS-CoV-2: 3.52, 95% CI: 3.19–3.86; IR influenza: 2.61, 95% CI: 1.10–4.12), and other ill-defined heart disease (IR SARS-CoV-2: 1.45, 95% CI: 1.24–1.67; IR influenza: 0.86, 95% CI: 0.00–1.72) (Table S4).

### 3.3. Relative Risk of Symptoms and Conditions at 90 Days after the Diagnosis Date

After applying 1: maximum PS matching by age group, gender, index month, year, and CCI, we selected 343 subjects from the SARS-CoV-2 and influenza pediatric cohorts and 1656 from the SARS-CoV-2 and influenza adult cohorts for analysis (Figures S2 and S3). We calculated the relative risk of symptoms and conditions at 1, 30 and 90 days after diagnosis for children and adults (Tables S5 and S6). 

**Figure 1:**
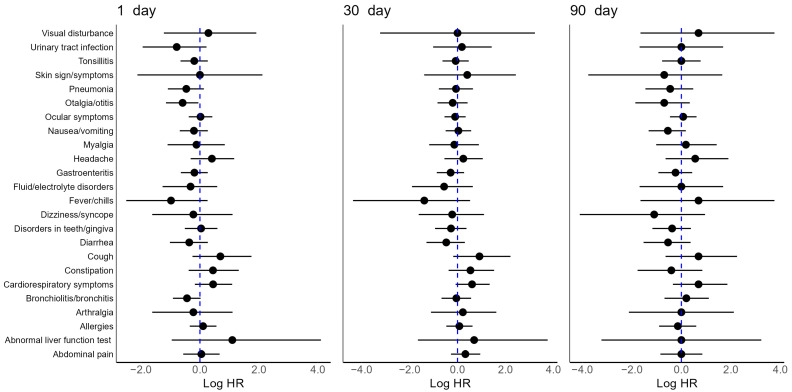
Relative risk of symptoms and conditions in PS matched children with SARS-CoV-2 vs. influenza at 1, 30, 90 days after the diagnosis date. Adjusted log HR (point) with 95% CI (solid line). The reference log HR = 0 (dotted blue line) represents no association (HR: hazard ratio, CI: confidence interval, PS: propensity score).

In children, the relative risk of symptoms and conditions in the SARS-CoV-2 cohort was generally not significantly different from the influenza cohort (Figure 1, Table S5). Only otalgia/otitis showed significantly reduced risk in SARS-CoV-2 children at 1 day following diagnosis, but this increase in risk disappeared at 30 and 90 days. 

**Figure 2:**
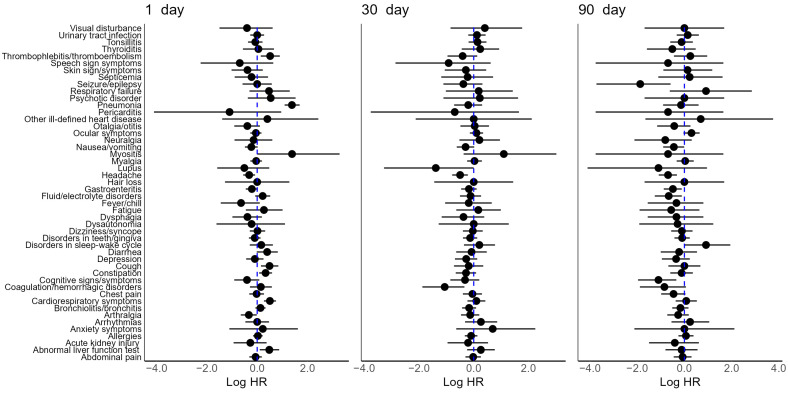
Relative risk of symptoms and conditions in PS matched adults with SARS-CoV-2 vs. influenza at 1, 30, 90 days after the diagnosis date. Adjusted log HR (point) with 95% CI (solid line). The reference log HR = 0 (dotted blue line) represents no association (HR: hazard ratio, CI: confidence interval, PS: propensity score).

For adults, at 1 day after the diagnosis date, SARS-CoV-2 adults showed an increased risk of abnormal liver function tests (HR 1.62, 95% CI: 1.11–2.40), cardiorespiratory symptoms (HR 1.66, 95% CI: 1.31–2.11), constipation (HR 1.40, 95% CI: 1.08–1.81), cough (HR 1.63, 95% CI: 1.16–2.32), thrombophlebitis/thromboembolism (HR 1.67, 95% CI: 1.15–2.46), and pneumonia (HR 3.98, 95% CI: 2.98–5.42) relative to influenza controls (Figure 2, Table S6). Also, a decreased risk for the following symptoms and conditions was observed in SARS-CoV-2 adults at 1 day: arthralgia (HR 0.72, 95% CI: 0.52–0.98) and headache (HR 0.73, 95% CI: 1.57–0.92) (Figure 2, Table S6). In contrast, at 30 and 90 days after the diagnosis date, SARS-CoV-2 adults showed a different symptom profile (Figure 2, Table S6). The symptoms and conditions that had increased risk at 1 day were no longer observed to be significantly elevated. At 30 days after the diagnosis date, SARS-CoV-2 adults showed lower risk of headache (HR 0.61, 95% CI: 0.46–0.81) and coagulation/hemorrhagic disorders (HR 0.35, 95% CI: 0.15–0.71) (Table S6). SARS-CoV-2 adults at 90 days only showed reduced risk for the following symptoms and conditions: cognitive signs/symptoms (HR 0.33, 95% CI: 0.14–0.71), headache (HR 0.50, 95% CI: 0.34–0.73), fluid/electrolyte imbalance (HR 0.51, 95% CI: 0.29–0.90), gastroenteritis (HR 0.61, 95% CI: 0.42–0.89), and seizure/epilepsy (HR 0.15, 95% CI: 0.02–0.56) (Figure 2, Table S6). 

### 3.4. Sensitivity Analyses

We performed sensitivity analyses to assess the robustness of the significant results found for SARS-CoV-2 infected adults at 90 days (Table S7). Similar to 1: maximum PS matched Cox regression, 1:1 PS matched Cox regression, 1:1 PS matched Poisson regression, and 1: maximum PS matched Poisson regression analyses all showed consistently reduced risk with similar *p*-values in SARS-CoV-2 adults for the following symptoms and conditions: cognitive signs/symptoms, headache, fluid/electrolyte imbalance, gastroenteritis, seizure/epilepsy (Table S7).

## 4. Discussion

### 4.1. Main Findings

In this nationwide retrospective observational study based on the Common Data Model, we aimed to characterize the sequelae of SARS-CoV-2 infection by assessing the relative risk of symptoms at 1, 30 and 90 days after diagnosis in both children and adults. We found that the symptom profile following SARS-CoV-2 infection evolves over time in ways that differ between children and adults. 

Adults who were diagnosed with SARS-CoV-2 showed an increased risk of outcomes like abnormal liver functions tests, cardiorespiratory symptoms, constipation, cough, thrombophlebitis/thromboembolism, and pneumonia at 1 day after diagnosis. However, these symptoms and conditions were no longer observed to have an increased risk at 30 and 90 days. Sensitivity analysis showed that the findings for SARS-CoV-2 infected adults at 90 days remained robust even when different ratios for PS matching and Poisson regression were used in the analysis. 

For children, we found that the risk of symptoms and conditions was not significantly elevated in those infected with SARS-CoV-2 compared to influenza controls during both the acute and post-acute periods after diagnosis. Only significantly decreased risk was observed for otalgia/otitis at 1 day after diagnosis. 

Persistent symptoms after viral infections are not novel; they have been previously reported in postviral syndromes following Middle East Respiratory Syndrome (MERS) and Severe Acute Respiratory Syndrome (SARS) infections [11]. It remains unclear whether the post-acute sequelae of SARS-CoV-2 are distinct from those that follow infections with other viruses. While some studies suggest that the post-acute sequelae of SARS-CoV-2 tend to be more severe compared to those of other viral infections such as influenza [13,20,24,25,26,27], other studies have reported otherwise, claiming that the post-acute sequelae of SARS-CoV-2 are comparable to those of other viral infections like influenza [28,29]. Our results, which show that the relative risk of symptoms in SARS-CoV-2 patients at 90 days after diagnosis is not significantly different from that of influenza controls, are consistent with the latter. 

The inconsistencies between our study and those that report a significantly elevated risk of post-acute sequelae may be due to several reasons. First, this difference may be attributed to the rollout of the SARS-CoV-2 vaccine in South Korea starting 26 February 2021 [30]. The vaccine rollout occurs during our cohort sampling period, which was from 1 January 2021 to 30 September 2021. By 17 May 2021, 7.3% of the entire Korean population had been vaccinated at least once [30]. It has been postulated that those vaccinated against SARS-CoV-2 experience fewer post-acute sequelae compared to those who are unvaccinated [31]. The protection provided through widespread vaccination may have reduced the risk of post-acute sequelae in our target cohort. Moreover, our study may have underreported the risk of sequelae in the target cohort because it did not consider the confounding effect of reinfections and variant types. Our study period overlaps with the third wave of the COVID-19 pandemic in Korea, defined as the period between 13 November 2020 and 6 July 2021 [32]. During this period, there were approximately 88,698 newly infected individuals in Seoul and surrounding metropolitan areas alone, which accounted for 69.6% of the total number of infections in the nation [32]. According to Kostka et al., reinfected individuals may experience increased risk of persistent symptoms compared to those who are infected for the first time [33]. Also, the third wave is characterized by the rise of variants (the alpha variant in December 2020, the beta and gamma variants in January 2021, the delta variant in April 2021) that appeared before the introduction of the delta and omicron variants [32]. Our study primarily focused on the initial waves of the pandemic, so the symptom profiles following infections with subsequent variants may differ [33]. Another reason for which SARS-CoV-2 patients may have had lower risk relative to controls may be attributed to changes in healthcare usage patterns. During the pandemic, hospital visits due to reasons other than COVID-19 decreased in Korea as well as in many other countries [34]. Thus, it is likely that those who made visits to the hospital for complaints other than COVID-19 were in more critical condition than those who made visits for complaints related to COVID-19 [28].

### 4.2. Previous Findings on the Sequelae of SARS-CoV-2 Infection in Adults

Whether morbidity is increased in adults after SARS-CoV-2 infection remains a topic of debate. While certain studies report an increased risk of conditions in the COVID-19 group, others maintain that this risk is not significantly elevated. Studies of the latter type include a population-based cohort study that claimed that hospitalization with COVID-19 was not associated with increased risk of outcomes such as cerebrovascular and cardiovascular disorders, neurological disorders, rheumatoid arthritis and mental health conditions relative to hospitalization with influenza prior to the pandemic or hospitalization with sepsis [28]. In the first 30 days following hospital discharge, SARS-CoV-2 infection was associated with an increased risk of venous thromboembolism, stroke, depression and anxiety, but these risks disappeared over time. This is consistent with our findings, which showed that symptoms like cough, cardiorespiratory symptoms, and abnormal liver function tests showed elevated risk in SARS-CoV-2 infected adults immediately following diagnosis but were resolved by 30 and 90 days. 

Moreover, a meta-analysis on persistent symptoms in hospitalized SARS-CoV-2 patients relative to non-SARS-CoV-2 hospitalized patients and nonhospitalized SARS-CoV-2 patients relative to healthy controls showed that many symptoms did not have higher incidence in SARS-CoV-2 patients [29]. In nonhospitalized SARS-CoV-2 patients compared to negative controls, most of the tested symptoms showed similar incidence rates. When hospitalized SARS-CoV-2 patients were compared to non-SARS-CoV-2 hospitalized patients, the former showed significantly lower odds of headache and sleep disorders, and similar odds of brain fog, anxiety and fatigue relative to controls. Also, while the risk of symptoms like anosmia, ageusia and brain fog was elevated in nonhospitalized SARS-CoV-2 patients relative to negative controls, the authors pointed out that these symptoms are nonspecific and frequently observed in other infectious diseases. Thus, the authors proposed that persistent symptoms after SARS-CoV-2 infection are due to factors like hospitalization rather than due to the virus itself. 

However, other studies reported an elevated risk of post-acute sequelae in SARS-CoV-2 infected patients relative to influenza controls. According to a study based on the hospital admissions data of the U.S. Department of Veteran Affairs Healthcare System, patients admitted to the hospital with SARS-CoV-2 showed higher rates of post-acute sequelae compared to those admitted with influenza prior to the pandemic [24]. These conditions included acute coronary syndrome, arrhythmias, acute kidney injury, chronic kidney disease, memory problems, and thromboembolic disease [24]. There was a risk gradient between outpatients and inpatients, with the greatest risk seen in those admitted to intensive care [24]. The authors concluded that there was increased risk of a wide range of symptoms as well as multiorgan involvement in SARS-CoV-2 patients relative to influenza controls [24]. 

According to a study by Liu et al., hospitalization with SARS-CoV-2 was associated with a higher risk of post-acute sequelae relative to hospitalization with influenza [25]. Those who were admitted for SARS-CoV-2 were at higher risk of prespecified outcomes such as abnormal breathing, abdominal symptoms, fatigue, and cognitive symptoms at 90–180 days after diagnosis [25]. SARS-CoV-2 patients also had an increased risk of all-cause emergency department visits, hospitalizations and mortality after discharge compared to those with influenza [25]. A systematic review showed that SARS-CoV-2 infection significantly increased the risk of fatigue, shortness of breath, neurological symptoms, memory problems and concentration problems at four weeks or more after infection compared to negative controls [13].

### 4.3. Previous Findings on the Sequelae of SARS-CoV-2 Infection in Children and Adolescents

According to the existing literature, there is also disagreement among studies on the burden of persistent symptoms in children and young people (CYP). There is a relative paucity of research on PASC in CYP, and studies tend to use different cutoff age values to define this group. Given that the Youth Protection Act in Korea defines a juvenile as any person under the age of 19, our study used 19 years as the cutoff age to define CYP, which was in line with other preexisting studies [35,36,37,38]. 

According to several studies, the burden of persistent symptoms appears to be high in CYP following infection with SARS-CoV-2 [39,40,41,42,43]. Some of these studies used controls [40,41,42,43], while others did not [39]. However, other studies, including ones that utilize electronic health records or claims data, have suggested that the burden of persistent symptoms in CYP may not be as high as previously claimed [3,44,45,46,47,48]. These differences may be attributed to factors like hospitalization status, age and gender [12,49,50,51]. A study by Haddad et al. showed that while the risk of moderate to severe symptoms was significantly higher in infected adults and adolescent girls (14–18 years), it was not significantly elevated in infected adolescent boys or children under 14 years of age. This suggests differences may arise between children and adolescents as well as between the sexes. Also, studies on hospitalized children tended to report a heavier burden of PASC compared to those on outpatient or community dwelling children [12,51]. 

Also, several meta-analyses were performed to assess the risk of long-term symptoms in SARS-CoV-2 cases compared to controls. According to a meta-analysis by Lopez-Leon that included a total of 21 studies and 80,071 children and adolescents, when compared to controls, infected children had a higher risk of persistent dyspnea, anosmia/ageusia, and fever [4]. In another meta-analysis by Behnood involving 22 studies with 23,131 CYP, the pooled risk difference in post-COVID cases was significantly higher for cognitive difficulties, headache, loss of smell, sore throat, and sore eyes compared to controls [35]. However, there was significant heterogeneity in these meta-analyses in terms of such factors as study design, study populations, the use of controls, follow-up time and assessment methods of exposure and outcome. 

### 4.4. Differences between the Clinical Presentation of PASC in Children and Adults

Overall, existing studies tend to depict a milder clinical presentation for PASC in CYP compared to adults. Persistent symptoms tend to be low in severity and prevalence, asymptomatic infections more frequent, and recovery time shorter in the younger patient population [11]. Among the studies that support this hypothesis is a matched cohort study based on national healthcare data covering 46% of the German population. Compared to negative control cohorts, both infected adults and children showed increased incidence rates of long-term health sequelae at least three months after diagnosis [20]. Also, the authors claimed that the incidence rates of these sequelae were consistently reduced in CYP compared to adults, although the overall burden may still be significant due to high infection rates [20]. 

Moreover, Mizrahi et al. conducted a nationwide cohort study based on electronic medical records that assessed the risk of 70 long COVID outcomes in children and adults relative to uninfected controls in the early phase (30–180 days) as well as in the late phase (180–360 days) [27]. According to this study, CYP who were 18 years of age and under had fewer outcomes following SARS-CoV-2 infection compared to adults. Also, most symptoms with elevated risk during the early period were resolved by the late period [27]. 

It has been hypothesized that differences in the pathophysiology of PASC underlie the distinctions in the clinical picture between CYP and adults. However, these biological pathways are yet unclear. Several mechanisms have been proposed, including differences in the state of the vascular endothelium and tendency towards clotting, ACE2 receptors and TMPRSS2 expression, preexisting immunity, immunosenescence, and the presence of comorbidities [52,53,54]. First, the endothelium of the vessels of older individuals tend to be more damaged, increasing the risk of thromboembolism and other complications [52]. Another mechanism involves the age-related differential expression of ACE2 receptors and TMPRSS2 [52,53,54]. Older individuals tend to express greater levels of ACE2, which have been implicated in enabling the entry of SARS-CoV-2 into cells. Moreover, adults are hypothesized to possess non-neutralizing human coronavirus (HCoV) antibodies that facilitate viral entry and replication [52]. Conversely, it has been proposed that children possess cross-reactive HCoV antibodies and T cells that exert a protective effect [52]. Lastly, the immature immune system of children may produce stronger innate immune response, clearing the virus more effectively, while eliciting a weaker adaptive immune response, leading to relatively lower levels of proinflammatory cytokines [52,54,55]. Further studies are needed to elucidate the molecular mechanisms behind the differences in the clinical presentation of PASC in CYP and adults. 

### 4.5. Strengths and Limitations

Our study has certain limitations. We relied on diagnostic codes from claims data for the assessment of exposure and outcomes. Since the entry of diagnostic codes is for the purpose of NHI reimbursement, there may be inconsistencies between these codes contained in the data source and the actual disease state of the patient. However, this is a limitation that applies to all research using claims data [15]. Also, there may be bias due to misclassification, as other viral infections may have been misclassified as influenza or SARS-CoV-2. Moreover, we were not able to assess the duration of symptoms and could only assess the incidence of certain symptoms at specific time points. Also, we used contemporaneous influenza controls even though rates of influenza declined during the COVID-19 pandemic [24]. With fewer influenza cases during the pandemic, estimating reliable relative risk becomes challenging. Confidence intervals for relative risk may widen, reducing the precision of the estimates. While we tried to use a non-contemporaneous influenza cohort as the control in our analysis, due to the temporal limitations of the sample database, we were unable to obtain a control cohort with a sufficient follow-up time. Moreover, we were not able to include variant types or vaccination status as covariates due to the lack of this information in the data source. The classification of cohorts by severity of initial illness was unsuccessful. Thus, we were not able to conduct sensitivity analyses according to several key factors that may influence the clinical picture of persistent symptoms.

To our understanding, this is the first study to investigate the differences in the post-acute symptoms and conditions of SARS-CoV-2 infection in the Korean population. The strengths of our study include its usage of a nationwide database and a study population sampled to ensure representativeness. Compared to analyses that are based on self-reported data or do not have controls, our study has greater reliability and validity and is less prone to bias such as recall bias. Additionally, our study is one of the first to use the OMOP HIRA COVID-19 database to implement a CDM-based distributed network research methodology for analyzing claims data from the pandemic. Our study demonstrates how this methodology can be effectively utilized to enable tailored observational research on healthcare data, addressing challenges related to patient privacy and data structure heterogeneity in research that uses electronic health records from various institutions.

## 5. Conclusions

Our study showed that adults diagnosed with SARS-CoV-2 face an elevated risk of symptoms and conditions immediately after diagnosis but experience a resolution of these sequelae by the 30 day mark. Furthermore, relative to influenza controls, SARS-CoV-2 infected CYP did not show any increased risk of sequelae throughout the 90 days of observation. Our study is limited by the lack of information on vaccination status and the type of viral variant, which was not included in the data source. Also, certain factors were not taken into account, such as the severity of the initial infection. Additionally, further studies that utilize different controls—including non-contemporaneous influenza and contemporaneous test-negative controls—will further enrich our understanding. This study not only provides insight into the sequelae of COVID-19 in the Korean population, but also sets the stage for future studies that utilize the methodology of OMOP-CDM, a powerful tool for epidemiologic investigation and interorganizational collaboration.

## Data Availability

HIRA does not make the data available but accepts analytic code from approved researchers. The approval process required to analyze OMOP-CDM converted claims data can be found at https://opendata.hira.or.kr/ (accessed on 9 April 2024).

## Supplementary Materials

The following supporting information can be downloaded at: https://www.mdpi.com/article/10.3390/jcm13102911/s1, Figure S1: Study Design; Figure S2: Attrition diagram for children; Figure S3: Attrition diagram for adults; Figure S4: Covariate balance plot after PS matching; Figure S5: Preference score distribution before propensity score adjustment for children; Figure S6: Preference score distribution before propensity score adjustment for adults; Figure S7: Kaplan–Meier plot for cough in children; Figure S8: Kaplan–Meier plot for cough in adults; Table S1: Baseline characteristics of children; Table S2: Baseline characteristics of adults; Table S3: Incidence proportion and rates of health outcomes in children with SARS-CoV-2 and influenza before PS matching; Table S4: Incidence proportion and rates of health outcomes in adults with SARS-CoV-2 and influenza at 90 days before PS matching; Table S5: Relative risk of symptoms and conditions in propensity score-matched children with SARS-CoV-2 vs. influenza at 1, 30, 90 days after the diagnosis date; Table S6: Relative risk of symptoms and conditions in propensity score-matched adults with SARS-CoV-2 vs. influenza at 1, 30, 90 days after the diagnosis date; Table S7: Sensitivity analysis of symptoms in adults at 90 days after diagnosis; Table S8: Target cohort (SARS-CoV-2) concept set; Table S9: Control cohort (Influenza) concept set; Table S10: Outcome (symptoms and conditions) concept set.
