# Supplementary material for: Symptoms and Conditions in Children and Adults up to 90 Days after SARS-CoV-2 Infection: A Retrospective Observational Study Utilizing the Common Data Model
Source: J Clin Med. 2024 May 15;13(10):2911. doi: 10.3390/jcm13102911 (PMC11122571; doi:10.3390/jcm13102911)
Supplement: Supplementary file 1 [file jcm-13-02911-s001.zip › jcm-2963752-supp1.pdf]

Supplementary Table S1. Baseline characteristics of children

	SARS-CoV-2 (N = 8879)	N (%)	Influenza (N = 345)	N (%)
**Age**				
	1-4	1444 (16.3)	1-4	145 (42.0)
	5-9	2108 (23.7)	5-9	61 (17.7)
	10-14	2184 (24.6)	10-14	62 (18.0)
	15-19	3143 (35.4)	15-19	77 (22.3)
**Gender**				
	Male	4835 (54.5)	Male	166 (48.1)
	Female	4044 (45.5)	Female	179 (51.9)

Supplementary Table S2. Baseline characteristics of adults

	SARS-CoV-2 (N = 51886)	N (%)	Influenza (N = 1672)	N (%)
**Age**				
	19-39	29785 (57.4)	19-39	439 (26.3)
	40-59	19593 (37.8)	40-59	477 (28.5)
	60-79	535 (1.0)	60-79	535 (32.0)
	80+	1973 (3.8)	80+	221 (13.2)
**Gender**				
	Male	26864 (51.8)	Male	766 (45.8)
	Female	25022 (48.2)	Female	906 (54.2)

Supplementary Table S3. Incidence proportion and rates of health outcomes in children with SARS-CoV-2 and influenza before propensity score matching

Symptoms/Conditions	SARS-CoV-2 child					Influenza child				
	Person count	Time at risk	Cases	Incidence proportion per 1k persons	Incidence rate per 1k years (95% CI)	Person count	Time at risk	Cases	Incidence proportion per 1k persons	Incidence rate per 1k years (95% CI)
**Abnormal liver function test**	8634	4962	46	5.33	9.27 (7.90-10.64)	335	253	2	5.97	7.91 (2.32-13.49)
**Fever/chill**	5958	3429	26	4.36	7.58 (6.10-9.07)	180	133	2	11.11	15.04 (4.40-25.67)
**Cardiorespiratory symptoms**	6425	3649	232	36.11	63.58 (59.40-67.75)	242	176	22	90.91	125.00 (98.35-151.65)
**Diarrhea**	7359	4211	117	15.90	27.78 (25.22-30.35)	264	191	15	56.82	78.53 (58.26-98.81)
**Allergies**	426	214	108	253.52	504.67 (456.11-553.24)	6	3	2	333.33	666.67 (195.26-1138.07)
**Skin sign/symptoms**	8455	4876	33	3.90	6.77 (5.59-7.95)	318	238	7	22.01	29.41 (18.30-40.53)
**Nausea/vomiting**	5143	2900	259	50.36	89.31 (83.76-94.86)	193	140	22	113.99	157.14 (123.64-190.65)
**Abdominal pain**	6141	3486	228	37.13	65.40 (61.07-69.74)	248	182	24	96.77	131.87 (104.95-158.79)
**Arthralgia**	7791	4445	166	21.31	37.35 (34.45-40.24)	315	235	6	19.05	25.53 (15.11-35.96)
**Constipation**	7602	4345	108	14.21	24.86 (22.46-27.25)	289	213	12	41.52	56.34 (40.07-72.60)
**Cough**	7661	4401	116	15.14	26.36 (23.91-28.80)	303	226	16	52.81	70.80 (53.10-88.50)
**Dizziness/syncope**	8337	4775	96	11.51	20.10 (18.05-22.16)	326	246	4	12.27	16.26 (8.13-24.39)
**Headache**	7038	4009	164	23.30	40.91 (37.71-44.10)	273	205	10	36.63	48.78 (33.35-64.21)
**Myalgia**	6590	3753	217	32.93	57.82 (53.90-61.75)	262	197	14	53.44	71.07 (52.07-90.06)
**Ocular symptoms**	3612	1993	233	64.51	116.91 (109.25-124.57)	157	108	26	165.61	240.74 (193.53-287.95)
**Otalgia/otitis**	5403	3057	192	35.54	62.81 (58.27-67.34)	158	116	14	88.61	120.69 (88.43-152.95)
**Visual disturbance**	8763	5051	20	2.28	3.96 (3.07-4.85)	336	255	2	5.95	7.84 (2.30-13.39)
**Fluid/electrolyte imbalance**	7723	4431	86	11.14	19.41 (17.32-21.50)	280	210	8	28.57	38.10 (24.63-51.56)
**Disorders of teeth/gingiva**	4999	2823	295	59.01	104.50 (98.41-110.58)	229	168	21	91.70	125.00 (97.72-152.28)
**Pneumonia**	6704	3837	147	21.93	38.31 (35.15-41.47)	231	167	20	86.58	119.76 (92.98-146.54)
**Bronchiolitis/bronchitis**	654	346	141	215.60	407.51 (373.20-441.83)	7	4	2	285.71	500.00 (146.45-853.55)

**Gastroenteritis**	2296	1279	216	94.08	168.88 (157.39-180.37)	58	37	9	155.17	243.24 (162.16-324.32)
**Tonsillitis**	3335	1853	301	90.25	162.44 (153.08-171.80)	115	79	21	182.61	265.82 (207.82-323.83)
**Urinary tract infection**	7739	4422	97	12.53	21.94 (19.71-24.16)	281	212	7	24.91	33.02 (20.54-45.50)

Supplementary Table S4. Incidence proportion and rates of health outcomes in adults with SARS-CoV-2 and influenza at 90 days before propensity score matching

Symptoms/conditions	SARS-CoV-2 adult					Influenza adult				
	Person count	Time at risk	Cases	Incidence proportion per 1k persons	Incidence rate per 1k years (95% CI)	Person count	Time at risk	Cases	Incidence proportion per 1k persons	Incidence rate per 1k years (95% CI)
**Hair loss**	50227	30513	183	3.64	6.00 (5.55-6.44)	1559	1160	5	4.31	4.31 (2.38-6.24)
**Chest pain**	39929	23740	1219	30.53	51.35 (49.88-52.82)	1073	786	50	63.61	63.61 (54.62-72.61)
**Abnormal liver function test**	46032	27777	566	12.30	20.38 (19.52-21.23)	1394	1033	27	26.14	26.14 (21.11-31.17)
**Anxiety symptoms**	50845	30929	59	1.16	1.91 (1.66-2.16)	1553	1157	2	1.73	1.73 (0.51-2.95)
**Fatigue**	49868	30271	199	3.99	6.57 (6.11-7.04)	1515	1129	17	15.06	15.06 (11.41-18.71)
**Fever/chill**	43460	26329	164	3.77	6.23 (5.74-6.72)	1053	781	7	6.65	8.96 (5.58-12.35)
**Cardiorespiratory symptoms**	33469	19892	1367	40.84	68.72 (66.86-70.58)	799	587	59	100.51	100.51 (87.43-113.60)
**Diarrhea**	46707	28250	461	9.87	16.32 (15.56-17.08)	1409	1038	26	25.05	25.05 (20.14-29.96)
**Respiratory failure**	51099	31097	38	0.74	1.22 (1.02-1.42)	1567	1167	6	5.14	5.14 (3.04-7.24)
**Allergies**	6856	3841	874	127.48	227.54 (219.85-235.24)	122	82	22	268.29	268.29 (211.09-325.49)
**Skin sign/symptoms**	49344	29926	288	5.84	9.62 (9.06-10.19)	1480	1090	18	16.51	16.51 (12.62-20.41)
**Nausea/vomiting**	35038	20797	1211	34.56	58.23 (56.56-59.90)	799	571	53	92.82	92.82 (80.07-105.57)
**Abdominal pain**	32610	19150	1659	50.87	86.63 (84.50-88.76)	789	559	74	132.38	132.38 (116.99-147.77)
**Arthralgia**	37349	22165	1325	35.48	59.78 (58.14-61.42)	1017	732	55	75.14	75.14 (65.01-85.27)
**Cognitive signs/symptoms**	48746	29439	289	5.93	9.82 (9.24-10.39)	1392	1035	18	17.39	17.39 (13.29-21.49)
**Constipation**	40957	24466	844	20.61	34.50 (33.31-35.68)	1066	784	52	66.33	66.33 (57.13-75.52)
**Cough**	41671	25201	668	16.03	26.51 (25.48-27.53)	1220	899	34	37.82	37.82 (31.33-44.31)
**Dizziness/syncope**	43161	25852	831	19.25	32.14 (31.03-33.26)	1156	845	44	52.07	52.07 (44.22-59.92)
**Dysphagia**	50826	30904	76	1.50	2.46 (2.18-2.74)	1532	1142	10	8.76	8.76 (5.99-11.53)
**Headache**	34356	20337	1379	40.14	67.81 (65.98-69.63)	861	613	75	122.35	122.35 (108.22-136.48)
**Myalgia**	24508	14322	1498	61.12	104.59 (101.89-107.30)	576	410	41	100.00	100.00 (84.38-115.62)
**Neuralgia**	49411	29942	284	5.75	9.49 (8.92-10.05)	1474	1094	13	11.88	11.88 (8.59-15.18)

**Ocular symptoms**	27625	16077	1603	58.03	99.71 (97.22-102.20)	684	493	47	95.33	95.33 (81.43-109.24)
**Otalgia/otitis**	45815	27652	566	12.35	20.47 (19.61-21.33)	1348	994	28	28.17	28.17 (22.85-33.49)
**Disorders in sleep-wake cycle**	47330	28604	383	8.09	13.39 (12.71-14.07)	1388	1027	17	16.55	16.55 (12.54-20.57)
**Speech sign symptoms**	50890	30951	52	1.02	1.68 (1.45-1.91)	1554	1158	4	3.45	3.45 (1.73-5.18)
**Visual disturbance**	50402	30630	88	1.75	2.87 (2.57-3.18)	1536	1142	7	6.13	6.13 (3.81-8.45)
**Myositis**	50444	30645	108	2.14	3.52 (3.19-3.86)	1541	1149	3	2.61	2.61 (1.10-4.12)
**Fluid/electrolyte imbalance**	44514	26814	626	14.06	23.35 (22.41-24.28)	1217	887	43	48.48	48.48 (41.09-55.87)
**Disorders of teeth/gingiva**	29486	17140	1969	66.78	114.88 (112.29-117.47)	836	597	69	115.58	115.58 (101.66-129.49)
**Other ill-defined heart disease**	50959	30988	45	0.88	1.45 (1.24-1.67)	1567	1166	1	0.86	0.86 (0.00-1.72)
**Acute kidney injury**	50378	30606	125	2.48	4.08 (3.72-4.45)	1504	1122	12	10.70	10.70 (7.61-13.78)
**Thrombophlebitis/thromboembolism**	47167	28518	466	9.88	16.34 (15.58-17.10)	1422	1045	38	36.36	36.36 (30.46-42.26)
**Pneumonia**	40524	24558	307	7.58	12.50 (11.79-13.21)	1221	908	24	26.43	26.43 (21.04-31.83)
**Bronchiolitis/bronchitis**	11625	6707	1223	105.20	182.35 (177.13-187.56)	189	129	39	302.33	302.33 (253.91-350.74)
**Gastroenteritis**	31352	18501	1495	47.68	80.81 (78.72-82.90)	749	527	74	140.42	140.42 (124.09-156.74)
**Arrhythmias**	48982	29666	263	5.37	8.87 (8.32-9.41)	1432	1062	18	16.95	16.95 (12.95-20.94)
**Dysautonomia**	50620	30768	85	1.68	2.76 (2.46-3.06)	1547	1150	11	9.57	9.57 (6.68-12.45)
**Depression**	43992	26401	649	14.75	24.58 (23.62-25.55)	1206	894	41	45.86	45.86 (38.70-53.02)
**Pericarditis**	51201	31157	18	0.35	0.58 (0.44-0.71)	1570	1169	2	1.71	1.71 (0.50-2.92)
**Psychotic disorder**	50773	30869	61	1.20	1.98 (1.72-2.23)	1552	1153	6	5.20	5.20 (3.08-7.33)
**Seizure epilepsy**	49589	30073	156	3.15	5.19 (4.77-5.60)	1469	1091	19	17.42	17.42 (13.42-21.41)
**Lupus**	50592	30757	92	1.82	2.99 (2.68-3.30)	1539	1145	6	5.24	5.24 (3.10-7.38)
**Thyroiditis**	48678	29480	298	6.12	10.11 (9.52-10.69)	1466	1086	15	13.81	13.81 (10.25-17.38)
**Septicemia**	50762	30881	101	1.99	3.27 (2.95-3.60)	1516	1126	14	12.43	12.43 (9.11-15.76)
**Tonsillitis**	29449	17640	1223	41.53	69.33 (67.35-71.31)	812	584	54	92.47	92.47 (79.88-105.05)
**Coagulation/hemorrhagic disorders**	49155	29771	304	6.18	10.21 (9.63-10.80)	1421	1059	25	23.61	23.61 (18.89-28.33)

**Urinary tract infection**	37228	22197	1295	34.79	58.34 (56.72-59.96)	952	689	49	71.12	71.12 (60.96-81.28)

Supplementary Table S5. Relative risk of symptoms and conditions in propensity score-matched children with SARS-CoV-2 vs. influenza at 1, 30, 90 days after the diagnosis date

	**1 day**		**30 days**		**90days**	
**Symptom/Condition**	HR (95% CI)	P-value	HR (95% CI)	P-value	HR (95% CI)	P-value
Abnormal liver function test	3.00 [0.38-60.62]	0.39	2.00 [0.19-43.01]	0.62	1.00 [0.04-25.27]	1.00
Fever/chill	0.38 [0.08-1.30]	0.16	0.25 [0.01-1.69]	0.27	2.00 [0.19-43.01]	0.62
Cardiorespiratory symptoms	1.56 [0.84-2.98]	0.17	1.83 [0.92-3.82]	0.09	2.00 [0.71-6.42]	0.22
Diarrhea	0.70 [0.36-1.31]	0.27	0.63 [0.27-1.36]	0.25	0.58 [0.22-1.45]	0.27
Allergies	1.11 [0.71-1.75]	0.65	1.08 [0.63-1.87]	0.78	0.87 [0.41-1.82]	0.71
Skin sign/symptoms	1.00 [0.12-8.33]	1.00	1.50 [0.25-11.39]	0.68	0.5 [0.02-5.22]	0.62
Nausea/vomiting	0.82 [0.50-1.31]	0.40	1.04 [0.61-1.77]	0.89	0.58 [0.27-1.20]	0.15
Abdominal pain	1.05 [0.57-1.95]	0.88	1.39 [0.76-2.58]	0.29	1.00 [0.43-2.34]	1.00
Arthralgia	0.80 [0.20-3.02]	0.75	1.25 [0.33-5.05]	0.75	1.00 [0.12-8.33]	1.00
Constipation	1.56 [0.68-3.73]	0.31	1.71 [0.69-4.61]	0.27	0.67 [0.17-2.33]	0.54
Cough	2.00 [0.78-5.74]	0.17	2.50 [0.84-9.11]	0.13	2.00 [0.53-9.48]	0.35
Dizziness/syncope	0.80 [0.20-3.02]	0.75	0.80 [0.20-3.02]	0.75	0.33 [0.02-2.60]	0.39
Headache	1.50 [0.73-3.20]	0.28	1.27 [0.58-2.87]	0.55	1.75 [0.53-6.68]	0.39
Myalgia	0.89 [0.33-2.32]	0.81	0.88 [0.31-2.44]	0.80	1.20 [0.36-4.16]	0.77
Ocular symptoms	1.02 [0.68-1.52]	0.92	0.90 [0.58-1.40]	0.66	1.08 [0.63-1.85]	0.79
Otalgia/otitis	**0.56 [0.32-0.95]**	**0.04**	0.82 [0.43-1.52]	0.53	0.50 [0.16-1.41]	0.22
Visual disturbance	1.33 [0.29-6.77]	0.72	1.00 [0.04-25.27]	1.00	2.00 [0.19-43.01]	0.62
Fluid/electrolyte imbalance	0.73 [0.28-1.80]	0.50	0.57 [0.15-1.89]	0.39	1.00 [0.19-5.40]	1.00
Disorders of teeth/gingiva	1.04 [0.60-1.81]	0.89	0.76 [0.39-1.45]	0.42	0.69 [0.31-1.47]	0.34
Pneumonia	0.63 [0.34-1.14]	0.14	0.94 [0.46-1.91]	0.86	0.64 [0.23-1.62]	0.36
Bronchiolitis/bronchitis	0.64 [0.40-1.02]	0.07	0.95 [0.51-1.76]	0.88	1.22 [0.51-3.03]	0.66

Gastroenteritis	0.83 [0.52-1.30]	0.42	0.75 [0.42-1.32]	0.32	0.79 [0.39-1.55]	0.5
Tonsillitis	0.83 [0.52-1.31]	0.42	0.93 [0.53-1.60]	0.78	1.00 [0.46-2.18]	1.00
Urinary tract infection	0.45 [0.14-1.25]	0.15	1.20 [0.36-4.16]	0.77	1.00 [0.19-5.40]	1.00

Supplementary Table S6. Relative risk of symptoms and conditions in propensity score-matched adults with SARS-CoV-2 vs. influenza at 1, 30, 90 days after the diagnosis date

	**1 day**		**30 days**		**90 days**	
Symptom/Condition	HR (95% CI)	P-value	HR (95% CI)	P-value	HR (95% CI)	P-value
Hair loss	1.00 [0.28-3.60]	1.00	1.00 [0.24-4.23]	1.00	1.00 [0.19-5.40]	1.00
Chest pain	0.98 [0.73-1.31]	0.88	0.95 [0.67-1.35]	0.79	0.63 [0.37-1.05]	0.08
Abnormal liver function test	**1.62 [1.11-2.40]**	**0.01**	1.30 [0.79-2.16]	0.31	0.88 [0.45-1.75]	0.73
Anxiety symptoms	1.25 [0.33-5.05]	0.75	2.00 [0.53-9.48]	0.35	1.00 [0.12-8.33]	1.00
Fatigue	1.31 [0.64-2.75]	0.47	1.18 [0.53-2.69]	0.69	0.57 [0.15-1.89]	0.39
Fever/chill	0.53 [0.24-1.11]	0.10	0.83 [0.35-1.93]	0.67	0.71 [0.21-2.24]	0.58
Cardiorespiratory symptoms	**1.66 [1.31-2.11]**	**0.00**	1.10 [0.8-1.53]	0.56	1.08 [0.69-1.70]	0.73
Diarrhea	1.49 [0.98-2.27]	0.06	0.92 [0.53-1.61]	0.78	0.80 [0.37-1.71]	0.57
Respiratory failure	1.60 [0.74-3.65]	0.25	1.20 [0.36-4.16]	0.77	2.50 [0.54-17.45]	0.30
Allergies	1.03 [0.85-1.24]	0.77	0.91 [0.72-1.14]	0.41	1.07 [0.78-1.47]	0.68
Skin sign/symptoms	0.68 [0.36-1.25]	0.22	0.75 [0.35-1.58]	0.46	1.14 [0.41-3.26]	0.80
Nausea/vomiting	0.79 [0.61-1.02]	0.07	0.74 [0.54-1.02]	0.07	0.64 [0.41-0.99]	0.05
Abdominal pain	0.94 [0.73-1.20]	0.61	0.98 [0.74-1.30]	0.89	0.93 [0.64-1.36]	0.70
Arthralgia	**0.72 [0.52-0.98]**	**0.04**	0.88 [0.63-1.23]	0.45	0.77 [0.48-1.20]	0.25
Cognitive signs/symptoms	0.67 [0.40-1.09]	0.11	0.73 [0.43-1.23]	0.24	**0.33 [0.14-0.71]**	**0.01**
Constipation	**1.40 [1.08-1.81]**	**0.01**	0.75 [0.52-1.09]	0.14	0.89 [0.55-1.43]	0.63
Cough	**1.63 [1.16-2.32]**	**0.01**	0.83 [0.49-1.42]	0.50	1.00 [0.51-1.97]	1.00
Dizziness/syncope	1.01 [0.75-1.37]	0.94	0.97 [0.67-1.39]	0.85	0.90 [0.57-1.41]	0.65
Dysphagia	0.68 [0.37-1.21]	0.20	0.69 [0.31-1.47]	0.34	0.71 [0.21-2.24]	0.58

Headache	**0.73 [0.57-0.92]**	**0.01**	**0.61 [0.46-0.81]**	**0.00**	**0.50 [0.34-0.73]**	**0.00**
Myalgia	0.96 [0.76-1.21]	0.73	1.03 [0.79-1.35]	0.84	1.03 [0.72-1.49]	0.85
Neuralgia	0.87 [0.41-1.82]	0.71	1.23 [0.59-2.60]	0.58	0.44 [0.12-1.36]	0.19
Ocular symptoms	0.95 [0.76-1.19]	0.65	1.10 [0.87-1.41]	0.42	1.35 [0.96-1.91]	0.09
Otalgia/otitis	0.68 [0.40-1.12]	0.13	1.04 [0.61-1.75]	0.90	0.65 [0.32-1.29]	0.23
Disorders in sleep-wake cycle	1.18 [0.75-1.87]	0.49	1.23 [0.70-2.17]	0.48	2.50 [1.02-7.01]	0.06
Speech sign symptoms	0.50 [0.11-1.90]	0.35	0.40 [0.06-1.86]	0.30	0.50 [0.02-5.22]	0.62
Visual disturbance	0.67 [0.22-1.85]	0.45	1.50 [0.43-5.87]	0.54	1.00 [0.19-5.40]	1.00
Myositis	4.00 [1.00-26.5]	0.10	3.00 [0.69-20.47]	0.20	0.50 [0.02-5.22]	0.62
Fluid/electrolyte imbalance	1.24 [0.92-1.68]	0.15	0.89 [0.60-1.31]	0.56	**0.51 [0.29-0.90]**	**0.02**
Disorders of teeth/gingiva	0.91 [0.72-1.14]	0.41	0.88 [0.68-1.14]	0.32	0.91 [0.66-1.26]	0.56
Other ill-defined heart disease	1.50 [0.25-11.39]	0.68	1.00 [0.12-8.33]	1.00	2.00 [0.19-43.01]	0.62
Acute kidney injury	0.76 [0.39-1.45]	0.42	0.81 [0.38-1.69]	0.58	0.67 [0.22-1.85]	0.45
Thrombophlebitis/thromboembolism	**1.67 [1.15-2.46]**	**0.01**	0.67 [0.38-1.14]	0.14	1.29 [0.64-2.63]	0.49
Pneumonia	**3.98 [2.98-5.42]**	**0.00**	0.82 [0.49-1.36]	0.44	0.87 [0.41-1.82]	0.71
Bronchiolitis/bronchitis	1.14 [0.93-1.39]	0.20	0.84 [0.65-1.09]	0.20	0.85 [0.60-1.19]	0.34
Gastroenteritis	0.81 [0.63-1.03]	0.09	0.84 [0.63-1.11]	0.22	**0.61 [0.42-0.89]**	**0.01**
Arrhythmias	1.00 [0.62-1.60]	1.00	1.30 [0.73-2.36]	0.38	1.27 [0.58-2.87]	0.55
Dysautonomia	0.8 [0.20-3.02]	0.75	1.00 [0.28-3.6]	1.00	0.75 [0.15-3.40]	0.72
Depression	0.91 [0.65-1.29]	0.60	0.76 [0.50-1.14]	0.19	0.70 [0.39-1.26]	0.24
Pericarditis	0.33 [0.02-2.60]	0.39	0.50 [0.02-5.22]	0.62	0.50 [0.02-5.22]	0.62
Psychotic disorder	1.71 [0.69-4.61]	0.27	1.25 [0.33-5.05]	0.75	1.00 [0.19-5.40]	1.00

Seizure/epilepsy	1.00 [0.56-1.79]	1.00	0.68 [0.33-1.37]	0.30	**0.15 [0.02-0.56]**	**0.02**
Lupus	0.60 [0.20-1.62]	0.33	0.25 [0.04-1]	0.10	0.33 [0.02-2.60]	0.39
Thyroiditis	1.05 [0.57-1.95]	0.88	1.27 [0.65-2.53]	0.50	0.60 [0.20-1.62]	0.33
Septicemia	0.8 [0.41-1.54]	0.51	0.8 [0.31-2.03]	0.64	1.25 [0.33-5.05]	0.75
Tonsillitis	0.93 [0.7-1.24]	0.61	1.15 [0.83-1.59]	0.41	0.89 [0.55-1.43]	0.63
Coagulation/hemorrhagic disorders	1.16 [0.75-1.79]	0.51	**0.35 [0.15-0.71]**	**0.01**	0.43 [0.15-1.07]	0.09
Urinary tract infection	1.00 [0.76-1.32]	1.00	1.12 [0.82-1.54]	0.47	1.15 [0.72-1.84]	0.56

Supplementary Table S7. Sensitivity analysis of symptoms in adults at 90 days after diagnosis

	HR (95% CI)	P-value
**Cognitive sign/symptom**		
1:1 PS, cox	0.3 (0.12-0.67)	$6.69*10^{-3}$
1:max PS, cox	0.3 (0.12-0.67)	$6.69*10^{-3}$
1:1 PS, poisson	0.27 (0.13-0.51)	$1.64*10^{-4}$
1:max PS, poisson	0.27 (0.13-0.51)	$1.64*10^{-4}$
**Headache**		
1:1 PS, cox	0.45 (0.30-0.66)	$8.69*10^{-5}$
1:max PS, cox	0.45 (0.30-0.66)	$8.69*10^{-6}$
1:1 PS, poisson	0.47 (0.34-0.64)	$2.83*10^{-6}$
1:max PS, poisson	0.47 (0.34-0.64)	$2.83*10^{-7}$
**Fluid/electrolyte imbalance**		
1:1 PS, cox	0.63 (0.36-1.06)	0.09
1:max PS, cox	0.63 (0.36-1.06)	0.09
1:1 PS, poisson	0.67 (0.43-1.04)	0.08
1:max PS, poisson	0.67 (0.43-1.04)	0.08
**Gastroenteritis**		
1:1 PS, cox	0.73 (0.51-1.04)	0.09
1:max PS, cox	0.73 (0.51-1.04)	0.09
1:1 PS, poisson	0.68 (0.51-0.92)	0.01
1:max PS, poisson	0.68 (0.51-0.92)	0.01
**Seizure/epilepsy**		
1:1 PS, cox	0.15 (0.02-0.56)	0.02
1:max PS, cox	0.15 (0.02-0.56)	0.02
1:1 PS, poisson	0.09 (0.01-0.30)	0.00
1:max PS, poisson	0.09 (0.01-0.30)	0.00

Supplementary Figure S1. Study Design

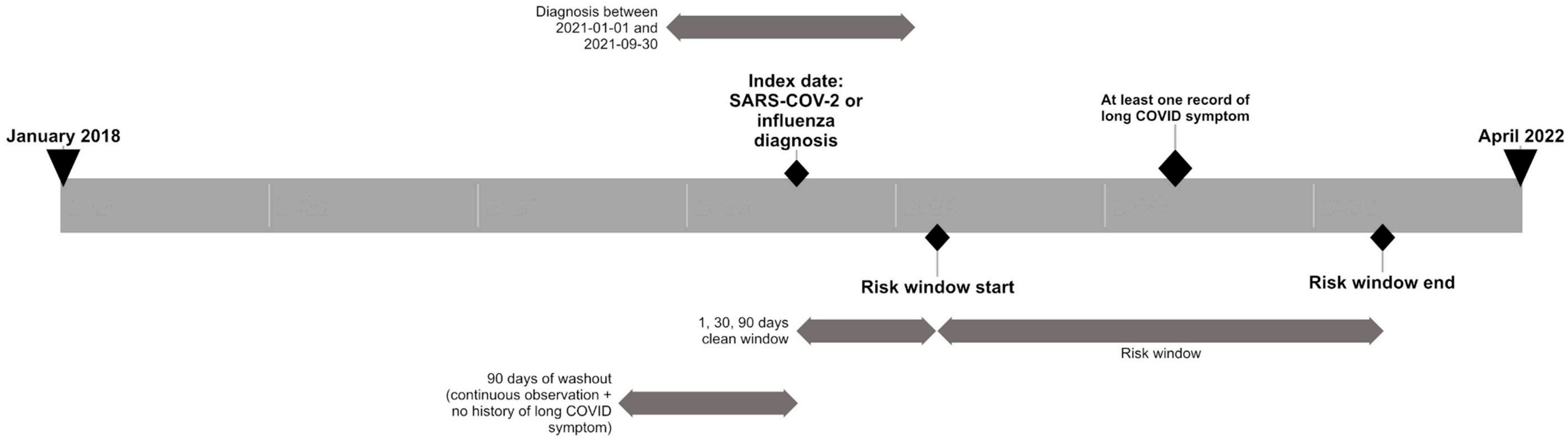

Supplementary Figure S2. Attrition diagram for children

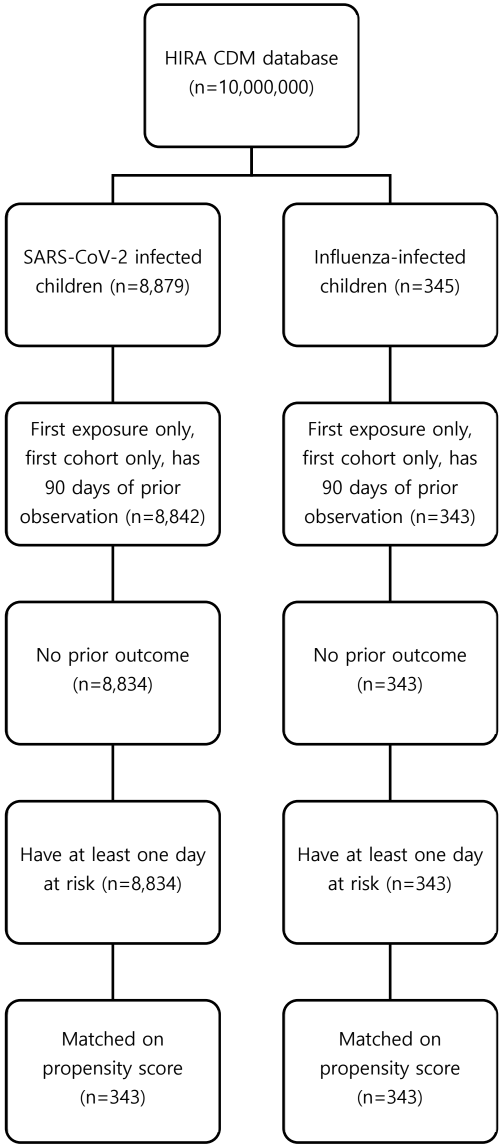

Supplementary Figure S3. Attrition diagram for adults

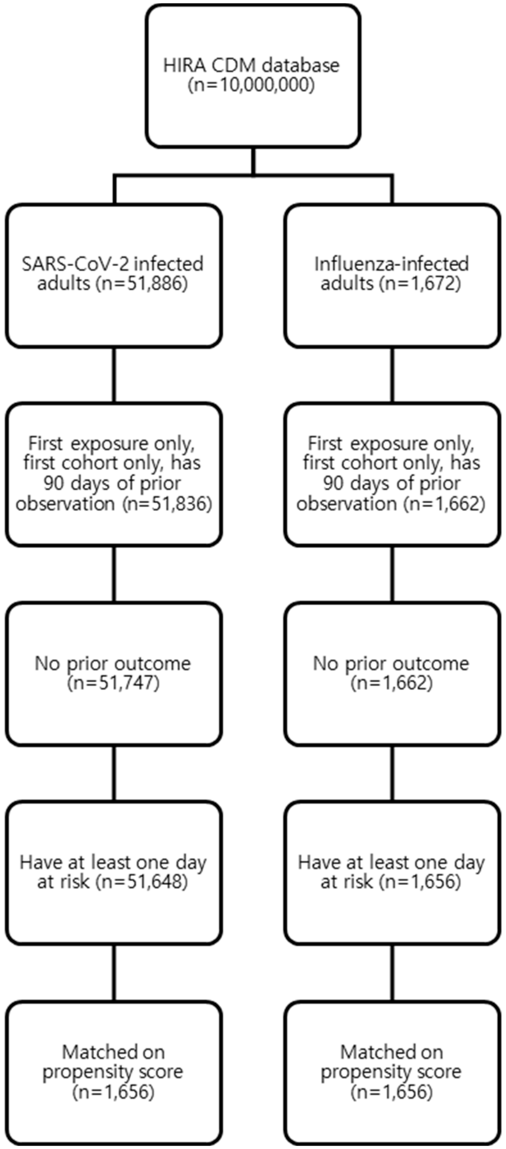

Supplementary Figure S4. Covariate balance plot after PS matching.

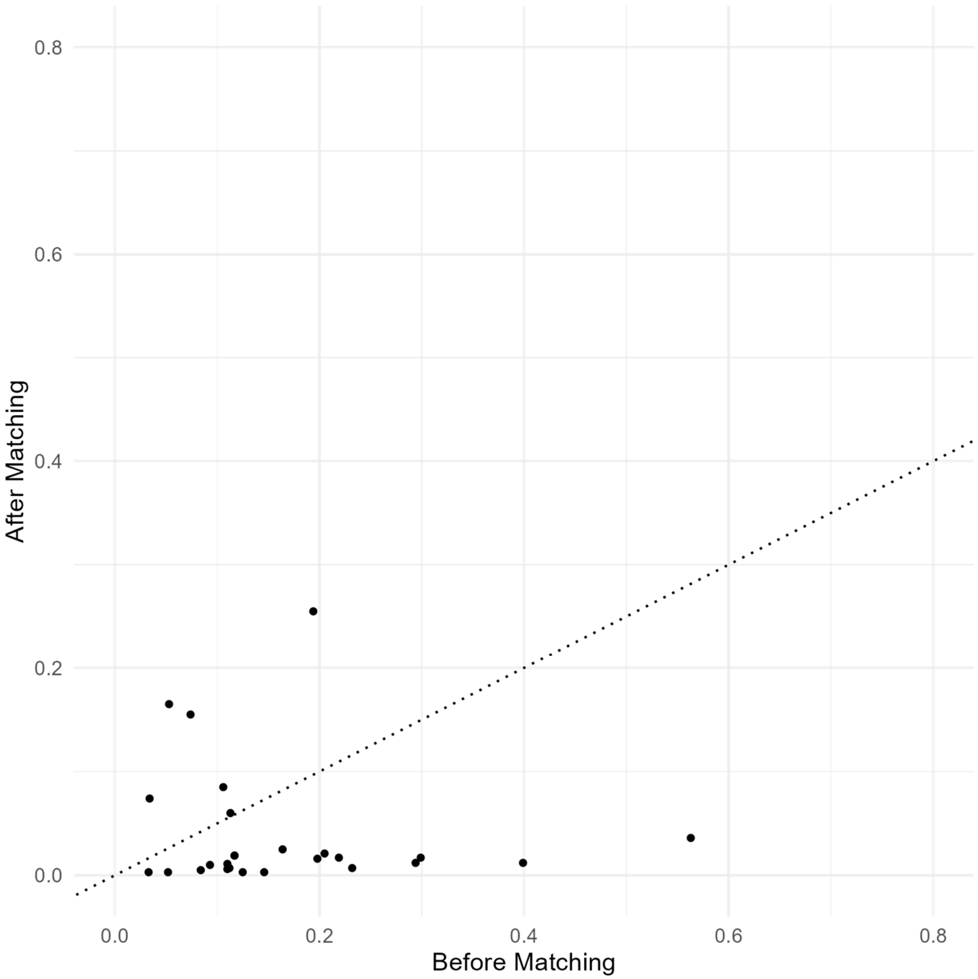

The covariate balance before and after PS matching is depicted for 26 covariates. Abbreviation: PS: propensity score.

Supplementary Figure S5. Preference score distribution before propensity score adjustment for children

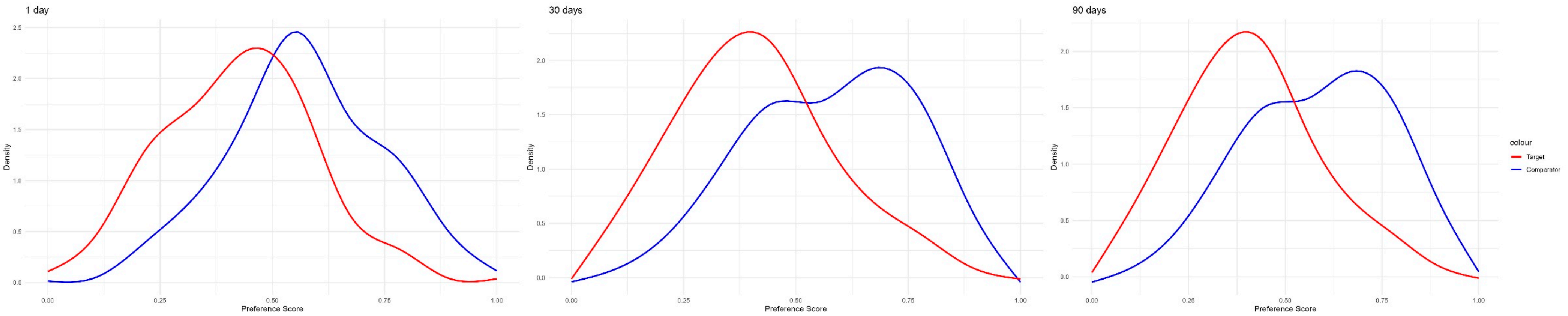

Supplementary Figure S6. Preference score distribution before propensity score adjustment for adults

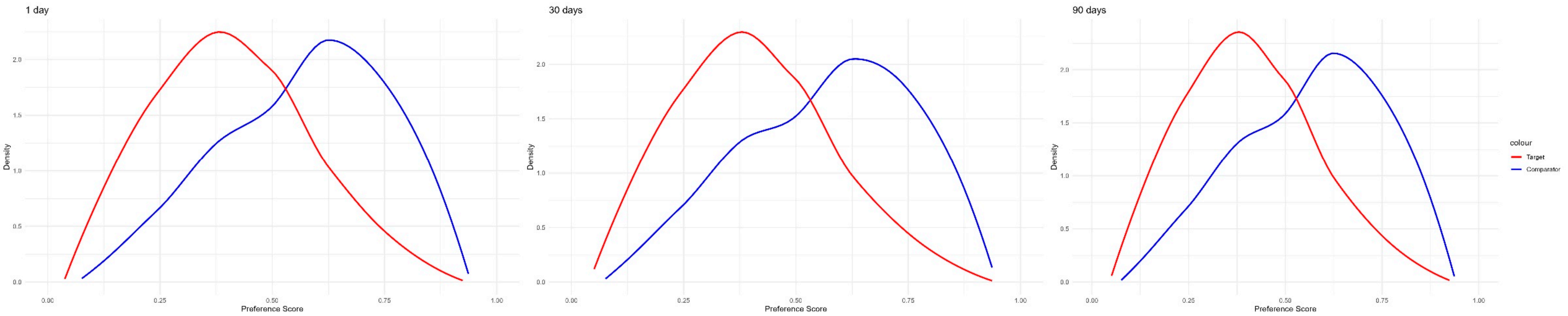

Supplementary Figure S7. Kaplan-Meier plot for cough in children

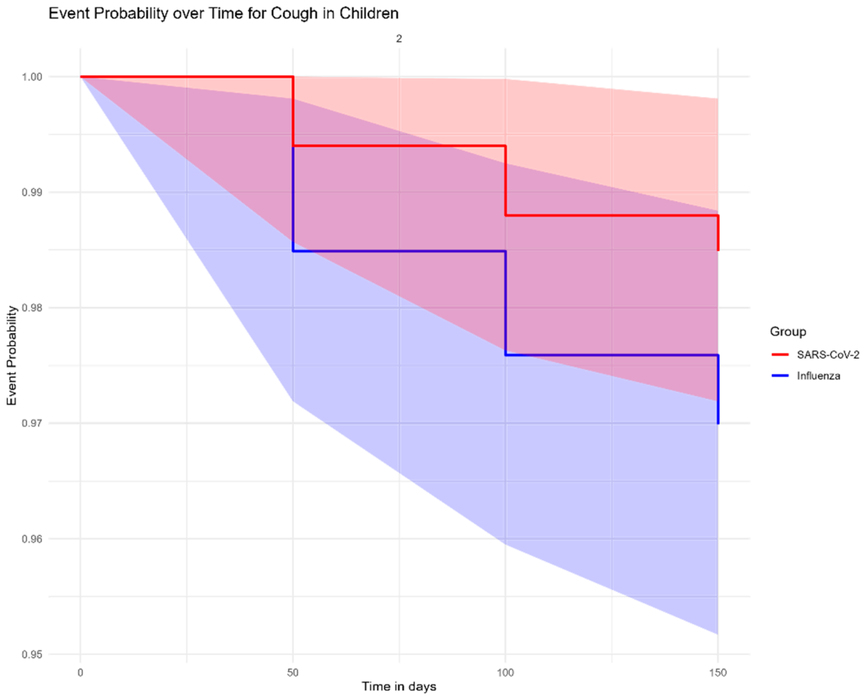

Kaplan-Meier plot showing the event probability of cough in SARS-CoV-2 and influenza infected children as a function of time. The y-axis represents the event probability and x-axis represents the time in days. The shaded area represents the 95% confidence interval.

Supplementary Figure S8. Kaplan-Meier plot for cough in adults

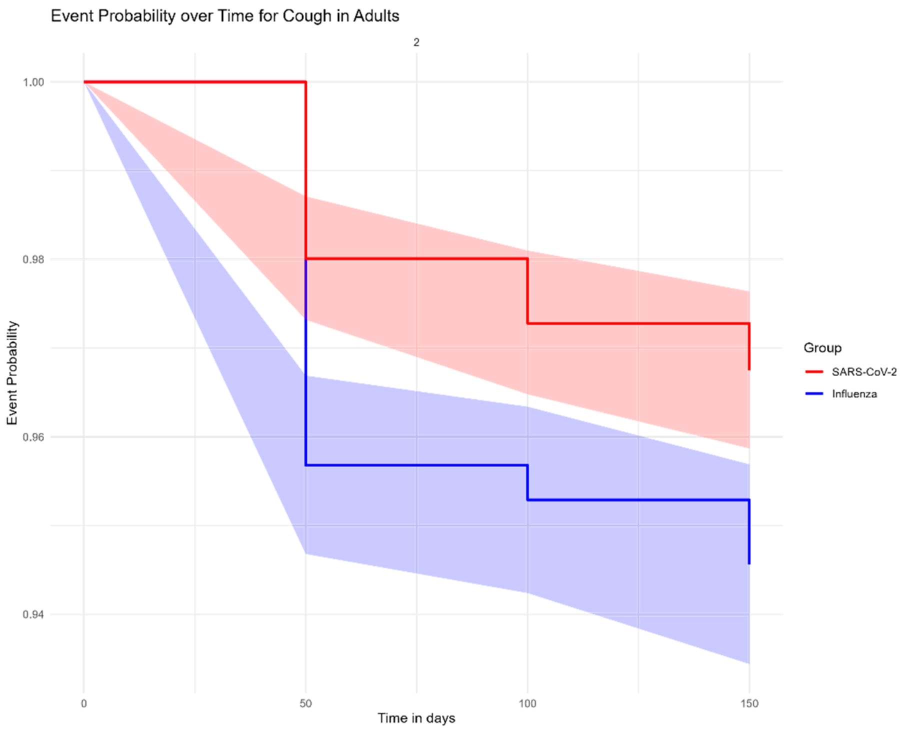

Kaplan-Meier plot showing the event probability of cough in SARS-CoV-2 and influenza infected adults as a function of time. The y-axis represents the event probability and x-axis represents the time in days. The shaded area represents the 95% confidence interval.
