# Supplementary material for: Symptoms and Conditions in Children and Adults up to 90 Days after SARS-CoV-2 Infection: A Retrospective Observational Study Utilizing the Common Data Model
Source: J Clin Med. 2024 May 15;13(10):2911. doi: 10.3390/jcm13102911 (PMC11122571; doi:10.3390/jcm13102911)
Supplement: Supplementary file 1 [file jcm-13-02911-s001.zip › jcm-2963752-supp2.pdf]

Supplementary Table S8. Target cohort (SARS-CoV-2) concept set

Concept Id	Concept Name	Domain	Vocabulary	Excluded	Descendants	Mapped
100065	Disease due to Coronaviridae	Condition	SNOMED	NO	NO	NO
37311061	Disease caused by 2019-nCoV	Condition	SNOMED	NO	NO	NO
439676	Coronavirus infection	Condition	SNOMED	NO	NO	NO
37311060	Suspected disease caused by 2019-nCoV	Observation	SNOMED	NO	YES	NO
704996	Patient meets COVID-19 laboratory diagnostic criteria	Observation	OMOP Extension	NO	YES	NO

Supplementary Table S9. Control cohort (Influenza) concept set

Concept Id	Concept Name	Domain	Vocabulary	Excluded	Descendants	Mapped
46273463	Upper respiratory tract infection due to Influenza	Condition	SNOMED	NO	YES	NO
36714388	Influenza caused by seasonal influenza virus	Condition	SNOMED	NO	YES	NO
4266367	Influenza	Condition	SNOMED	NO	YES	NO

Supplementary Table S10. Outcome (symptoms and conditions) concept set

■ Change in smell taste

Concept Id	Concept Name	Domain	Vocabulary	Excluded	Descendants	Mapped
436235	Taste sense altered	Condition	SNOMED	NO	NO	NO
43530714	Sensory disorder of smell and/or taste	Condition	SNOMED	NO	NO	NO
46270715	Parosmia	Condition	SNOMED	NO	NO	NO
4185711	Loss of sense of smell	Condition	SNOMED	NO	NO	NO

■ Hair loss

Concept Id	Concept Name	Domain	Vocabulary	Excluded	Descendants	Mapped
140173	Telogen effluvium	Condition	SNOMED	NO	NO	NO
4266794	Scarring alopecia	Condition	SNOMED	NO	NO	NO
4239312	Ophiasis	Condition	SNOMED	NO	NO	NO
4031164	Non-scarring alopecia	Condition	SNOMED	NO	NO	NO
4339092	Male pattern alopecia	Condition	SNOMED	NO	NO	NO
4066853	Drug-induced androgenic alopecia	Condition	SNOMED	NO	NO	NO
4313298	Anagen effluvium	Condition	SNOMED	NO	NO	NO
4312756	Alopecia universalis	Condition	SNOMED	NO	NO	NO
4056343	Alopecia totalis	Condition	SNOMED	NO	NO	NO
4099746	Alopecia mucinosa	Condition	SNOMED	NO	NO	NO
141933	Alopecia areata	Condition	SNOMED	NO	NO	NO

■ Chest pain

Concept Id	Concept Name	Domain	Vocabulary	Excluded	Descendants	Mapped
134159	Precordial pain	Condition	SNOMED	NO	NO	NO
4168213	Chest pain on breathing	Condition	SNOMED	NO	NO	NO
77670	Chest pain	Condition	SNOMED	NO	NO	NO

■ Abnormal liver function test

Concept Id	Concept Name	Domain	Vocabulary	Excluded	Descendants	Mapped
438878	Liver function tests abnormal	Condition	SNOMED	NO	NO	NO
436238	Elevated levels of transaminase & lactic acid dehydrogenase	Condition	SNOMED	NO	NO	NO

■ Anxiety symptoms

Concept Id	Concept Name	Domain	Vocabulary	Excluded	Descendants	Mapped
4251306	Stress	Condition	SNOMED	NO	NO	NO
4168212	Restlessness and agitation	Condition	SNOMED	NO	NO	NO
4238682	Physical AND emotional exhaustion state	Condition	SNOMED	NO	NO	NO
4085332	Physical aggression	Condition	SNOMED	NO	NO	NO
4269314	Mental state finding	Condition	SNOMED	NO	NO	NO
4168681	Irritability and anger	Condition	SNOMED	NO	NO	NO
4195585	Hostile behavior	Condition	SNOMED	NO	NO	NO
4169106	Feeling unhappy	Condition	SNOMED	NO	NO	NO
436817	Feeling nervous	Condition	SNOMED	NO	NO	NO

■ Fatigue

Concept Id	Concept Name	Domain	Vocabulary	Excluded	Descendants	Mapped
4202045	Postviral fatigue syndrome	Condition	SNOMED	NO	NO	NO
439926	Malaise and fatigue	Condition	SNOMED	NO	NO	NO

■ Fever/chills

Concept Id	Concept Name	Domain	Vocabulary	Excluded	Descendants	Mapped
4164645	Fever with chills	Condition	SNOMED	NO	NO	NO
437663	Fever	Condition	SNOMED	NO	NO	NO

■ Cardiorespiratory symptoms

Concept Id	Concept Name	Domain	Vocabulary	Excluded	Descendants	Mapped
314754	Wheezing	Condition	SNOMED	NO	NO	NO

444070	Tachycardia	Condition	SNOMED	NO	NO	NO
253321	Stridor	Condition	SNOMED	NO	NO	NO
432427	Severe birth asphyxia	Condition	SNOMED	NO	NO	NO
317109	Respiratory arrest	Condition	SNOMED	NO	NO	NO
315531	Respiration intermittent	Condition	SNOMED	NO	NO	NO
134159	Precordial pain	Condition	SNOMED	NO	NO	NO
315078	Palpitations	Condition	SNOMED	NO	NO	NO
4298207	Mouth breathing	Condition	SNOMED	NO	NO	NO
434759	Mild to moderate birth asphyxia	Condition	SNOMED	NO	NO	NO
314171	Low blood pressure reading	Condition	SNOMED	NO	NO	NO
316814	Hyperventilation	Condition	SNOMED	NO	NO	NO
261687	Hemoptysis	Condition	SNOMED	NO	NO	NO
316822	Heart murmur	Condition	SNOMED	NO	NO	NO
4114164	Finding of heart sounds	Condition	SNOMED	NO	NO	NO
141693	Elevated blood-pressure reading without diagnosis of hypertension	Observation	SNOMED	NO	NO	NO
312437	Dyspnea	Condition	SNOMED	NO	NO	NO
4180628	Disorder of body system	Condition	SNOMED	NO	NO	NO
438555	Cyanosis	Condition	SNOMED	NO	NO	NO
254761	Cough	Condition	SNOMED	NO	NO	NO
4169095	Bradycardia	Condition	SNOMED	NO	NO	NO
4184996	Birth asphyxia	Condition	SNOMED	NO	NO	NO
4279614	Asphyxiation	Observation	SNOMED	NO	NO	NO
433596	Abnormal sputum	Condition	SNOMED	NO	NO	NO
4262562	Abnormal heart beat	Condition	SNOMED	NO	NO	NO
4305080	Abnormal breathing	Condition	SNOMED	NO	NO	NO
4305577	Abnormal blood pressure	Condition	SNOMED	NO	NO	NO

■ Respiratory failure

Concept Id	Concept Name	Domain	Vocabulary	Excluded	Descendants	Mapped
4256228	Respiratory failure	Conditio	SNOME	NO	NO	NO

		n	D			
317109	Respiratory arrest	Condition	SNOMED	NO	NO	NO
37395564	Hypoxemic respiratory failure	Condition	SNOMED	NO	NO	NO
46271074	Hypercapnic respiratory failure	Condition	SNOMED	NO	NO	NO
314971	Chronic respiratory failure	Condition	SNOMED	NO	NO	NO
4177862	Chronic hypoxemic respiratory failure	Condition	SNOMED	NO	NO	NO
4140438	Chronic hypercapnic respiratory failure	Condition	SNOMED	NO	NO	NO
319049	Acute respiratory failure	Condition	SNOMED	NO	NO	NO
46271075	Acute hypoxemic respiratory failure	Condition	SNOMED	NO	NO	NO
46273719	Acute hypercapnic respiratory failure	Condition	SNOMED	NO	NO	NO

■ Diarrhea

Concept Id	Concept Name	Domain	Vocabulary	Excluded	Descendants	Mapped
36715842	Non-infective neonatal diarrhea	Condition	SNOMED	NO	NO	NO
4320650	Non-infective diarrhea	Condition	SNOMED	NO	NO	NO
4153574	Neonatal diarrhea	Condition	SNOMED	NO	NO	NO
198337	Infectious diarrheal disease	Condition	SNOMED	NO	NO	NO
80141	Functional diarrhea	Condition	SNOMED	NO	NO	NO
4134607	Diarrheal disorder	Condition	SNOMED	NO	NO	NO
196523	Diarrhea	Condition	SNOMED	NO	NO	NO

■ Allergies

Concept Id	Concept Name	Domain	Vocabulary	Excluded	Descendants	Mapped
139100	Vibratory urticaria	Condition	SNOMED	NO	NO	NO
4305500	Vasomotor rhinitis	Condition	SNOMED	NO	NO	NO
4065355	Urticaria due to cold and heat	Condition	SNOMED	NO	NO	NO
139900	Urticaria	Condition	SNOMED	NO	NO	NO
141651	Stevens-Johnson syndrome	Condition	SNOMED	NO	NO	NO
4014199	Solar urticaria	Condition	SNOMED	NO	NO	NO
4280726	Seasonal allergic rhinitis	Condition	SNOMED	NO	NO	NO
4320791	Rhinitis	Condition	SNOMED	NO	NO	NO

4080921	Polymorphous light eruption	Condition	SNOMED	NO	NO	NO
4308570	Phototoxic drug eruption	Observation	SNOMED	NO	NO	NO
4197654	Photoallergic drug eruption	Condition	SNOMED	NO	NO	NO
4216188	Nummular eczema	Condition	SNOMED	NO	NO	NO
4008574	Noninfectious dermatosis of eyelid	Condition	SNOMED	NO	NO	NO
4032899	Non-bullous erythema multiforme	Condition	SNOMED	NO	NO	NO
4066819	Localized skin eruption caused by drug and medicament	Condition	SNOMED	NO	NO	NO
4066820	Ingestion dermatitis due to food	Condition	SNOMED	NO	NO	NO
45766714	Inflammatory dermatosis	Condition	SNOMED	NO	NO	NO
140803	Idiopathic urticaria	Condition	SNOMED	NO	NO	NO
4064036	Generalized skin eruption caused by drug and medicament	Condition	SNOMED	NO	NO	NO
434219	Food anaphylaxis	Condition	SNOMED	NO	NO	NO
132702	Erythema multiforme	Condition	SNOMED	NO	NO	NO
132983	Dermatographic urticaria	Condition	SNOMED	NO	NO	NO
135032	Dermatitis due to substances taken internally	Condition	SNOMED	NO	NO	NO
4023944	Contact urticaria	Condition	SNOMED	NO	NO	NO
444375	Contact dermatitis due to plants, except food	Condition	SNOMED	NO	NO	NO
379019	Conjunctivitis	Condition	SNOMED	NO	NO	NO
376425	Chronic non-suppurative otitis media	Condition	SNOMED	NO	NO	NO
138501	Cholinergic urticaria	Condition	SNOMED	NO	NO	NO
4066727	Besnier's prurigo	Condition	SNOMED	NO	NO	NO
4066470	Berloque dermatitis	Condition	SNOMED	NO	NO	NO
133834	Atopic dermatitis	Condition	SNOMED	NO	NO	NO
80488	Arthropathy associated with a hypersensitivity reaction	Condition	SNOMED	NO	NO	NO
432791	Angioedema	Condition	SNOMED	NO	NO	NO
441202	Anaphylaxis	Condition	SNOMED	NO	NO	NO
139902	Allergic urticaria	Condition	SNOMED	NO	NO	NO
256439	Allergic rhinitis due to pollen	Condition	SNOMED	NO	NO	NO
257007	Allergic rhinitis	Condition	SNOMED	NO	NO	NO
43530807	Allergic disposition	Condition	SNOMED	NO	NO	NO

46270315	Allergic contact dermatitis due to metal	Condition	SNOMED	NO	NO	NO
4064030	Allergic contact dermatitis due to food in contact with the skin	Condition	SNOMED	NO	NO	NO
4064029	Allergic contact dermatitis due to dye	Condition	SNOMED	NO	NO	NO
4064028	Allergic contact dermatitis due to drug in contact with skin	Condition	SNOMED	NO	NO	NO
4066735	Allergic contact dermatitis due to cosmetic	Condition	SNOMED	NO	NO	NO
4066471	Allergic contact dermatitis due to adhesive	Condition	SNOMED	NO	NO	NO
45770891	Allergic contact dermatitis caused by plant material	Condition	SNOMED	NO	NO	NO
46269791	Allergic contact dermatitis caused by chemical	Condition	SNOMED	NO	NO	NO
4031019	Allergic contact dermatitis	Condition	SNOMED	NO	NO	NO
441488	Adverse reaction to substance	Observation	SNOMED	NO	NO	NO
4105886	Adverse reaction	Condition	SNOMED	NO	NO	NO
374948	Acute secretory otitis media	Condition	SNOMED	NO	NO	NO
4290728	Acute effect of ultraviolet radiation on normal skin	Condition	SNOMED	NO	NO	NO

■ Skin sign symptoms

Concept Id	Concept Name	Domain	Vocabulary	Excluded	Descendants	Mapped
443257	Swelling / lump finding	Condition	SNOMED	NO	NO	NO
438252	Spontaneous ecchymosis	Condition	SNOMED	NO	NO	NO
137682	Skin sensation disturbance	Condition	SNOMED	NO	NO	NO
141960	Skin finding	Condition	SNOMED	NO	NO	NO
4144409	Pale - symptom	Condition	SNOMED	NO	NO	NO
42872381	Numbness and tingling sensation of skin	Condition	SNOMED	NO	NO	NO
4168701	Localized swelling, mass and lump, upper limb	Condition	SNOMED	NO	NO	NO
4166126	Localized swelling, mass and lump, trunk	Condition	SNOMED	NO	NO	NO
4166125	Localized swelling, mass and lump, neck	Condition	SNOMED	NO	NO	NO
4171920	Localized swelling, mass and lump, multiple sites	Condition	SNOMED	NO	NO	NO
4171919	Localized swelling, mass and lump, lower limb	Condition	SNOMED	NO	NO	NO
4161682	Hypesthesia	Condition	SNOMED	NO	NO	NO
4031840	Hyperesthesia	Conditi	SNOME	NO	NO	NO

		on	D			
318566	Flushing	Condition	SNOMED	NO	NO	NO
140842	Changes in skin texture	Condition	SNOMED	NO	NO	NO
4155909	Anesthesia of skin	Condition	SNOMED	NO	NO	NO

■ Nausea/vomiting

Concept Id	Concept Name	Domain	Vocabulary	Excluded	Descendants	Mapped
441408	Vomiting	Condition	SNOMED	NO	NO	NO
4312477	Projectile vomiting	Condition	SNOMED	NO	NO	NO
27674	Nausea and vomiting	Condition	SNOMED	NO	NO	NO
31967	Nausea	Condition	SNOMED	NO	NO	NO

■ Abdominal pain

Concept Id	Concept Name	Domain	Vocabulary	Excluded	Descendants	Mapped
4306292	Upper abdominal pain	Condition	SNOMED	NO	NO	NO
198263	Right upper quadrant pain	Condition	SNOMED	NO	NO	NO
193322	Right lower quadrant pain	Condition	SNOMED	NO	NO	NO
4168216	Pelvic and perineal pain	Condition	SNOMED	NO	NO	NO
4091513	Passing flatus	Condition	SNOMED	NO	NO	NO
4116811	Pain of truncal structure	Condition	SNOMED	NO	NO	NO
4182562	Lower abdominal pain	Condition	SNOMED	NO	NO	NO
194175	Left upper quadrant pain	Condition	SNOMED	NO	NO	NO
195083	Left lower quadrant pain	Condition	SNOMED	NO	NO	NO
197988	Generalized abdominal pain	Condition	SNOMED	NO	NO	NO
4241033	Acute abdomen	Condition	SNOMED	NO	NO	NO
200215	Abdominal rigidity	Condition	SNOMED	NO	NO	NO
200219	Abdominal pain	Condition	SNOMED	NO	NO	NO

■ Anorexia

Concept Id	Concept Name	Domain	Vocabulary	Excluded	Descendants	Mapped
4233612	Psychogenic loss of appetite	Condition	SNOMED	NO	NO	NO
4102962	Non-organic loss of appetite	Condition	SNOMED	NO	NO	NO
4103560	Non-organic infant feeding disturbance	Conditi	SNOME	NO	NO	NO

		on	D			
4250314	Feeding disorder of infancy OR early childhood	Condition	SNOMED	NO	NO	NO
439002	Eating disorder	Condition	SNOMED	NO	NO	NO

■ Arthralgia

Concept Id	Concept Name	Domain	Vocabulary	Excluded	Descendants	Mapped
78232	Shoulder joint pain	Condition	SNOMED	NO	NO	NO
761651	Pain of joint of right lower leg	Condition	SNOMED	NO	NO	NO
759910	Pain of joint of right foot	Condition	SNOMED	NO	NO	NO
761652	Pain of joint of left lower leg	Condition	SNOMED	NO	NO	NO
765269	Pain of joint of left foot	Condition	SNOMED	NO	NO	NO
765249	Pain of joint of bilateral lower legs	Condition	SNOMED	NO	NO	NO
37017182	Joint pain of pelvic region	Condition	SNOMED	NO	NO	NO
759905	Joint pain in right hand	Condition	SNOMED	NO	NO	NO
759906	Joint pain in left hand	Condition	SNOMED	NO	NO	NO
77074	Joint pain	Condition	SNOMED	NO	NO	NO
78234	Hand joint pain	Condition	SNOMED	NO	NO	NO
37209669	Bilateral pain of joint of hands	Condition	SNOMED	NO	NO	NO
79106	Arthralgia of the pelvic region and thigh	Condition	SNOMED	NO	NO	NO

78508	Arthralgia of the ankle and/or foot	Condition	SNOMED	NO	NO	NO
4116168	Ankle joint pain	Condition	SNOMED	NO	NO	NO
45757286	Acute arthralgia of knee	Condition	SNOMED	NO	NO	NO
4116165	Acromioclavicular joint pain	Condition	SNOMED	NO	NO	NO

■ Balance problems

Concept Id	Concept Name	Domain	Vocabulary	Excluded	Descendants	Mapped
441417	Incoordination	Condition	SNOMED	NO	NO	NO
36714126	Difficulty walking	Condition	SNOMED	NO	NO	NO
4114624	Coordination problem	Condition	SNOMED	NO	NO	NO
4096848	Ataxic gait	Observation	SNOMED	NO	NO	NO
437584	Ataxia	Condition	SNOMED	NO	NO	NO
437643	Abnormal gait	Observation	SNOMED	NO	NO	NO

■ Chilblains

Concept Id	Concept Name	Domain	Vocabulary	Excluded	Descendants	Mapped
141456	Chilblains	Condition	SNOMED	NO	NO	NO

■ Cognitive signs and symptoms

Concept Id	Concept Name	Domain	Vocabulary	Excluded	Descendants	Mapped
4024707	Perception AND/OR perception disturbance	Condition	SNOMED	NO	NO	NO
4297400	Mild cognitive disorder	Condition	SNOMED	NO	NO	NO
4293175	Mental state, behavior and/or psychosocial function finding	Condition	SNOMED	NO	NO	NO
4268911	Disorientated	Condition	SNOMED	NO	NO	NO
4047120	Disorders of attention and motor control	Condition	SNOMED	NO	NO	NO
440213	Consciousness and/or awareness finding	Condition	SNOMED	NO	NO	NO
4162723	Cognitive function finding	Condition	SNOMED	NO	NO	NO

404806<br>15	Cognitive disorder	Conditi<br>on	SNOM<br>ED	NO	NO	NO
132342	Apraxia	Conditi<br>on	SNOM<br>ED	NO	NO	NO
439147	Amnesia	Conditi<br>on	SNOM<br>ED	NO	NO	NO
417313<br>6	Agnosia	Conditi<br>on	SNOM<br>ED	NO	NO	NO

■ Constipation

Concept Id	Concept Name	Domain	Vocabulary	Excluded	Descendants	Mapped
79061	Slow transit constipation	Condition	SNOMED	NO	NO	NO
201905	Constipation by outlet obstruction	Condition	SNOMED	NO	NO	NO
75860	Constipation	Condition	SNOMED	NO	NO	NO

■ Cough

Concept Id	Concept Name	Domain	Vocabulary	Excluded	Descendants	Mapped
254761	Cough	Condition	SNOMED	NO	NO	NO

■ Dizziness/syncope

Concept Id	Concept Name	Domain	Vocabulary	Excluded	Descendants	Mapped
439383	Vertigo	Condition	SNOMED	NO	NO	NO
4206148	Syncope and collapse	Condition	SNOMED	NO	NO	NO
135360	Syncope	Condition	SNOMED	NO	NO	NO
78162	Peripheral vertigo	Condition	SNOMED	NO	NO	NO
433316	Dizziness and giddiness	Condition	SNOMED	NO	NO	NO
4223938	Dizziness	Condition	SNOMED	NO	NO	NO

■ Dysphagia

Concept Id	Concept Name	Domain	Vocabulary	Excluded	Descendants	Mapped
36716717	Functional dysphagia	Condition	SNOMED	NO	NO	NO
4254223	Esophageal dysphagia	Condition	SNOMED	NO	NO	NO
31317	Dysphagia	Condition	SNOMED	NO	NO	NO
44788979	Developmental dysphagia	Condition	SNOMED	NO	NO	NO
44788952	Acquired dysphagia	Condition	SNOMED	NO	NO	NO

■ Headache

Concept Id	Concept Name	Domain	Vocabulary	Excluded	Descendants	Mapped
376382	Tension-type headache	Condition	SNOMED	NO	NO	NO
378735	Migraine without aura	Condition	SNOMED	NO	NO	NO
381549	Migraine with aura	Condition	SNOMED	NO	NO	NO
318736	Migraine	Condition	SNOMED	NO	NO	NO
375527	Headache disorder	Condition	SNOMED	NO	NO	NO
378253	Headache	Condition	SNOMED	NO	NO	NO
381278	Cluster headache	Condition	SNOMED	NO	NO	NO

■ Myalgia

Concept Id	Concept Name	Domain	Vocabulary	Excluded	Descendants	Mapped
4121935	Myalgia/myositis -pelvis/thigh	Condition	SNOMED	NO	NO	NO
4121598	Myalgia/myositis - upper arm	Condition	SNOMED	NO	NO	NO
4121597	Myalgia/myositis - shoulder	Condition	SNOMED	NO	NO	NO
4125937	Myalgia/myositis - multiple	Condition	SNOMED	NO	NO	NO
4125939	Myalgia/myositis - lower leg	Condition	SNOMED	NO	NO	NO
4121934	Myalgia/myositis - hand	Condition	SNOMED	NO	NO	NO
4125938	Myalgia/myositis - forearm	Condition	SNOMED	NO	NO	NO
4126064	Myalgia/myositis - ankle/foot	Condition	SNOMED	NO	NO	NO
442752	Muscle pain	Condition	SNOMED	NO	NO	NO

■ Neuralgia

Concept Id	Concept Name	Domain	Vocabulary	Excluded	Descendants	Mapped
373852	Neuralgia	Condition	SNOMED	NO	NO	NO

■ Ocular symptoms

Concept Id	Concept Name	Domain	Vocabulary	Excluded	Descendants	Mapped
4208666	Viral eye infection	Condition	SNOMED	NO	NO	NO
380038	Viral conjunctivitis	Condition	SNOMED	NO	NO	NO

379031	Pain in eye	Conditio n	SNOMED	NO	NO	NO
4174302	Neonatal infection of the eye	Conditio n	SNOMED	NO	NO	NO
380397	Keratoconjunctivitis	Conditio n	SNOMED	NO	NO	NO
444207	Inflammatory disorder of the eye	Conditio n	SNOMED	NO	NO	NO
4103653	Infective conjunctivitis	Conditio n	SNOMED	NO	NO	NO
4188177	Immune-mediated conjunctivitis	Conditio n	SNOMED	NO	NO	NO
4134613	Eye infection	Conditio n	SNOMED	NO	NO	NO
4036620	Dry eyes	Conditio n	SNOMED	NO	NO	NO
373499	Disorder of eye region	Conditio n	SNOMED	NO	NO	NO
375252	Disorder of eye	Conditio n	SNOMED	NO	NO	NO
379019	Conjunctivitis	Conditio n	SNOMED	NO	NO	NO
4335888	Atopic conjunctivitis	Conditio n	SNOMED	NO	NO	NO
43021807	Allergic conjunctivitis	Conditio n	SNOMED	NO	NO	NO
4132556	Acute disease of eye	Conditio n	SNOMED	NO	NO	NO
376707	Acute conjunctivitis	Conditio n	SNOMED	NO	NO	NO
380111	Acute atopic conjunctivitis	Conditio n	SNOMED	NO	NO	NO

■ Otalgia/otitis

Conc ept Id	Concept Name	Dom ain	Voca bular y	Excl uded	Desce ndants	Ma ppe d
7649 95	Suppurative otitis media of right ear	Cond ition	SNO MED	NO	NO	NO
7601 69	Suppurative otitis media of left ear	Cond ition	SNO MED	NO	NO	NO
4288 879	Subacute nonsuppurative otitis media	Cond ition	SNO MED	NO	NO	NO
7641 18	Spontaneous rupture of right tympanic membrane co-occurrent and due to acute suppurative otitis media	Cond ition	SNO MED	NO	NO	NO
7653 11	Spontaneous rupture of left tympanic membrane co-occurrent and due to acute suppurative otitis media	Cond ition	SNO MED	NO	NO	NO
7600 40	Spontaneous rupture of bilateral tympanic membranes co-occurrent and due to acute suppurative otitis media	Cond ition	SNO MED	NO	NO	NO
7653 23	Serous otitis media of right ear	Cond ition	SNO MED	NO	NO	NO
7601 68	Serous otitis media of left ear	Cond ition	SNO MED	NO	NO	NO
7601 26	Serous otitis media of bilateral ears	Cond ition	SNO MED	NO	NO	NO
4214 270	Serous otitis media	Cond ition	SNO MED	NO	NO	NO
7602	Rupture of right tympanic membrane due to otitis media	Cond	SNO	NO	NO	NO

04		ition	MED			
4626 9815	Recurrent acute suppurative otitis media with spontaneous rupture of ear drum	Cond ition	SNO MED	NO	NO	NO
4335 736	Recurrent acute suppurative otitis media	Cond ition	SNO MED	NO	NO	NO
7601 81	Recurrent acute serous otitis media of right middle ear	Cond ition	SNO MED	NO	NO	NO
7601 42	Recurrent acute serous otitis media of left middle ear	Cond ition	SNO MED	NO	NO	NO
7602 10	Recurrent acute otitis media of right ear	Cond ition	SNO MED	NO	NO	NO
7601 67	Recurrent acute otitis media of left ear	Cond ition	SNO MED	NO	NO	NO
7649 85	Recurrent acute otitis media of bilateral ears	Cond ition	SNO MED	NO	NO	NO
4110 794	Recurrent acute otitis media	Cond ition	SNO MED	NO	NO	NO
4336 165	Recurrent acute non-suppurative otitis media	Cond ition	SNO MED	NO	NO	NO
3767 12	Purulent otitis media	Cond ition	SNO MED	NO	NO	NO
7601 61	Perforation of tympanic membrane of left ear due to otitis media	Cond ition	SNO MED	NO	NO	NO
7653 09	Perforation of tympanic membrane of bilateral ears due to otitis media	Cond ition	SNO MED	NO	NO	NO
4576 9847	Perforation of tympanic membrane due to otitis media	Cond ition	SNO MED	NO	NO	NO
3781 60	Otorrhea	Cond ition	SNO MED	NO	NO	NO
7602 03	Otitis media of right ear	Cond ition	SNO MED	NO	NO	NO
7653 14	Otitis media of left ear	Cond ition	SNO MED	NO	NO	NO
7601 19	Otitis media of bilateral ears	Cond ition	SNO MED	NO	NO	NO
3723 28	Otitis media	Cond ition	SNO MED	NO	NO	NO
3807 33	Otalgia	Cond ition	SNO MED	NO	NO	NO
4170 137	Non-suppurative otitis media	Cond ition	SNO MED	NO	NO	NO
4208 784	Infective otitis media	Cond ition	SNO MED	NO	NO	NO
3781 61	Disorder of ear	Cond ition	SNO MED	NO	NO	NO
3813 01	Chronic serous otitis media	Cond ition	SNO MED	NO	NO	NO
4096 343	Bleeding from ear	Cond ition	SNO MED	NO	NO	NO
4108 522	Bilateral suppurative otitis media	Cond ition	SNO MED	NO	NO	NO
3668 5071	Bilateral recurrent acute serous otitis media of middle ears	Cond ition	SNO MED	NO	NO	NO
4576 6332	Bacterial otitis media	Cond ition	SNO MED	NO	NO	NO
4095 693	Allergic otitis media	Cond ition	SNO MED	NO	NO	NO
3752 81	Acute suppurative otitis media without spontaneous rupture of ear drum	Cond ition	SNO MED	NO	NO	NO

381028	Acute suppurative otitis media with spontaneous rupture of ear drum	Condition	SNOMED	NO	NO	NO
760183	Acute suppurative otitis media of right ear	Condition	SNOMED	NO	NO	NO
760144	Acute suppurative otitis media of left ear	Condition	SNOMED	NO	NO	NO
760039	Acute suppurative otitis media of bilateral ears	Condition	SNOMED	NO	NO	NO
379028	Acute suppurative otitis media due to another disease	Condition	SNOMED	NO	NO	NO
439264	Acute suppurative otitis media	Condition	SNOMED	NO	NO	NO
760182	Acute serous otitis media of right ear	Condition	SNOMED	NO	NO	NO
760143	Acute serous otitis media of left ear	Condition	SNOMED	NO	NO	NO
760038	Acute serous otitis media of bilateral ears	Condition	SNOMED	NO	NO	NO
374948	Acute secretory otitis media	Condition	SNOMED	NO	NO	NO
4111238	Acute right otitis media	Condition	SNOMED	NO	NO	NO
760034	Acute recurrent allergic otitis media of bilateral middle ears	Condition	SNOMED	NO	NO	NO
4150372	Acute otitis media	Condition	SNOMED	NO	NO	NO
760178	Acute non-suppurative otitis media of right ear	Condition	SNOMED	NO	NO	NO
760140	Acute non-suppurative otitis media of left ear	Condition	SNOMED	NO	NO	NO
760031	Acute non-suppurative otitis media of bilateral ears	Condition	SNOMED	NO	NO	NO
375827	Acute non-suppurative otitis media - serous	Condition	SNOMED	NO	NO	NO
4110795	Acute left otitis media	Condition	SNOMED	NO	NO	NO
4110796	Acute bilateral otitis media	Condition	SNOMED	NO	NO	NO

■ Disorders in sleep-wake cycle

Concept Id	Concept Name	Domain	Vocabulary	Excluded	Descendants	Mapped
377535	Sleep walking disorder	Condition	SNOMED	NO	NO	NO
4232324	Sleep terror disorder	Condition	SNOMED	NO	NO	NO
4102985	Nonorganic insomnia	Condition	SNOMED	NO	NO	NO
374905	Non-organic sleep disorder	Condition	SNOMED	NO	NO	NO
377266	Non-organic disorder of the sleep-wake schedule	Condition	SNOMED	NO	NO	NO
4170260	Nightmares	Condition	SNOMED	NO	NO	NO

■ Speech/sign symptoms

Concept Id	Concept Name	Domain	Vocabulary	Excluded	Descendants	Mapped
4128827	Voice production finding	Condition	SNOMED	NO	NO	NO
4168556	Hypernasality and hyponasality	Condition	SNOMED	NO	NO	NO
4024717	Dyslexia AND/OR speech dysfunction	Condition	SNOMED	NO	NO	NO
435642	Disturbance in speech	Condition	SNOMED	NO	NO	NO
4114720	Difficulty talking	Condition	SNOMED	NO	NO	NO
40481303	Aphonia	Condition	SNOMED	NO	NO	NO
4024716	Aphasia, agnosia, dyslexia AND/OR apraxia	Condition	SNOMED	NO	NO	NO

■ Visual disturbance

Concept Id	Concept Name	Domain	Vocabulary	Excluded	Descendants	Mapped
4134440	Visual system disorder	Condition	SNOMED	NO	NO	NO
4265433	Visual impairment	Condition	SNOMED	NO	NO	NO
4275889	Visual hallucinations	Condition	SNOMED	NO	NO	NO
377286	Visual field defect	Condition	SNOMED	NO	NO	NO
374034	Visual disturbance	Condition	SNOMED	NO	NO	NO
381001	Subjective visual disturbance	Condition	SNOMED	NO	NO	NO
4201690	Finding of visual field	Condition	SNOMED	NO	NO	NO
4038502	Eye / vision finding	Condition	SNOMED	NO	NO	NO
257293	Disorder of visual pathways	Condition	SNOMED	NO	NO	NO
373474	Diplopia	Condition	SNOMED	NO	NO	NO

■ Acute respiratory distress syndrome

Concept Id	Concept Name	Domain	Vocabulary	Excluded	Descendants	Mapped
4195694	Acute respiratory distress syndrome	Condition	SNOMED	NO	NO	NO

■ Myocarditis

Concept Id	Concept Name	Domain	Vocabulary	Excluded	Descendants	Mapped
4231274	Viral myocarditis	Conditio	SNOMED	NO	NO	NO

		n				
4087608	Viral carditis	Condition	SNOMED	NO	NO	NO
319825	Rheumatic heart disease	Condition	SNOMED	NO	NO	NO
4331309	Myocarditis due to infectious agent	Condition	SNOMED	NO	NO	NO
314383	Myocarditis	Condition	SNOMED	NO	NO	NO
317208	Meningococcal carditis	Condition	SNOMED	NO	NO	NO
4143969	Isolated (Fiedler's) myocarditis	Condition	SNOMED	NO	NO	NO
320127	Bacterial myocarditis	Condition	SNOMED	NO	NO	NO
321578	Acute rheumatic myocarditis	Condition	SNOMED	NO	NO	NO
312653	Acute myocarditis	Condition	SNOMED	NO	NO	NO

■ Myositis

Concept Id	Concept Name	Domain	Vocabulary	Excluded	Descendants	Mapped
4231274	Viral myocarditis	Condition	SNOMED	NO	NO	NO
4087608	Viral carditis	Condition	SNOMED	NO	NO	NO
319825	Rheumatic heart disease	Condition	SNOMED	NO	NO	NO
4331309	Myocarditis due to infectious agent	Condition	SNOMED	NO	NO	NO
314383	Myocarditis	Condition	SNOMED	NO	NO	NO
317208	Meningococcal carditis	Condition	SNOMED	NO	NO	NO
4143969	Isolated (Fiedler's) myocarditis	Condition	SNOMED	NO	NO	NO
320127	Bacterial myocarditis	Condition	SNOMED	NO	NO	NO
321578	Acute rheumatic myocarditis	Condition	SNOMED	NO	NO	NO
312653	Acute myocarditis	Condition	SNOMED	NO	NO	NO

■ Fluid/electrolyte disorders

Concept Id	Concept Name	Domain	Vocabulary	Excluded	Descendants	Mapped
441536	Mixed acid-base balance disorder	Condition	SNOMED	NO	NO	NO
436670	Metabolic disease	Condition	SNOMED	NO	NO	NO
37311319	Hypovolemia	Condition	SNOMED	NO	NO	NO
437833	Hypokalemia	Condition	SNOMED	NO	NO	NO
435515	Hypo-osmolality and or	Conditio	SNOME	NO	NO	NO

	hyponatremia	n	D			
434004	Hypervolemia	Condition	SNOMED	NO	NO	NO
441829	Hyperosmolality and or hypernatremia	Condition	SNOMED	NO	NO	NO
434610	Hyperkalemia	Condition	SNOMED	NO	NO	NO
441830	Disorder of fluid AND/OR electrolyte	Condition	SNOMED	NO	NO	NO
438730	Alkalosis	Condition	SNOMED	NO	NO	NO
435517	Acidosis	Condition	SNOMED	NO	NO	NO

■ Disorders in teeth/gingiva

Concept Id	Concept Name	Domain	Vocabulary	Excluded	Descendants	Mapped
4061788	Total anodontia of permanent and deciduous teeth	Condition	SNOMED	NO	NO	NO
4122116	Tooth eruption disorder	Condition	SNOMED	NO	NO	NO
135852	Teething syndrome	Condition	SNOMED	NO	NO	NO
4197943	Taurodontism	Condition	SNOMED	NO	NO	NO
37399393	Symptomatic periapical periodontitis	Condition	SNOMED	NO	NO	NO
40636815	Supernumerary teeth	Condition	SNOMED	NO	NO	NO
4028685	Sensitive dentin	Condition	SNOMED	NO	NO	NO
4033617	Reversible pulpitis	Condition	SNOMED	NO	NO	NO
138181	Radicular cyst	Condition	SNOMED	NO	NO	NO
437589	Pulpitis	Condition	SNOMED	NO	NO	NO
435854	Pulp degeneration	Condition	SNOMED	NO	NO	NO
435570	Pulp and periapical tissue disease	Condition	SNOMED	NO	NO	NO
440758	Posteruptive color change of tooth	Condition	SNOMED	NO	NO	NO
134398	Periodontal disease	Condition	SNOMED	NO	NO	NO
133803	Periapical abscess with sinus tract	Condition	SNOMED	NO	NO	NO
4025325	Periapical abscess	Condition	SNOMED	NO	NO	NO
441329	Pathological resorption of tooth	Condition	SNOMED	NO	NO	NO
438794	Partial congenital absence of teeth	Condition	SNOMED	NO	NO	NO
4122221	Odontogenic cyst	Condition	SNOMED	NO	NO	NO

4001755	Occlusal wear of teeth	Condition	SNOMED	NO	NO	NO
437316	Non-odontogenic developmental cyst of jaw	Condition	SNOMED	NO	NO	NO
437590	Necrosis of the pulp	Condition	SNOMED	NO	NO	NO
45757361	Mottled teeth	Condition	SNOMED	NO	NO	NO
4138525	Microdontia	Condition	SNOMED	NO	NO	NO
4325100	Macrodontia	Condition	SNOMED	NO	NO	NO
4173420	Juvenile periodontitis	Condition	SNOMED	NO	NO	NO
4032727	Irreversible pulpitis	Condition	SNOMED	NO	NO	NO
4195567	Irradiated enamel	Condition	SNOMED	NO	NO	NO
4123726	Impacted tooth	Condition	SNOMED	NO	NO	NO
442576	Hypercementosis	Condition	SNOMED	NO	NO	NO
45757251	Hereditary disturbances in tooth structure	Condition	SNOMED	NO	NO	NO
134386	Gingival recession	Condition	SNOMED	NO	NO	NO
4182983	Gingival enlargement	Condition	SNOMED	NO	NO	NO
132344	Gingival and periodontal disease	Condition	SNOMED	NO	NO	NO
4055511	Gingival and edentulous alveolar ridge lesion associated with trauma	Condition	SNOMED	NO	NO	NO
4222116	Gemination of teeth	Condition	SNOMED	NO	NO	NO
4068529	Fusion of teeth	Condition	SNOMED	NO	NO	NO
438783	Erosion of teeth	Condition	SNOMED	NO	NO	NO
4292864	Enamel pearls	Condition	SNOMED	NO	NO	NO
4131814	Enamel hypoplasia	Condition	SNOMED	NO	NO	NO
4024688	Embedded teeth	Condition	SNOMED	NO	NO	NO
4159157	Disorder of tooth development	Condition	SNOMED	NO	NO	NO
201603	Disorder of teeth AND/OR supporting structures	Condition	SNOMED	NO	NO	NO
141050	Disorder of hard tissues of teeth	Condition	SNOMED	NO	NO	NO
4050079	Dislocation of tooth	Condition	SNOMED	NO	NO	NO
4147380	Dental fluorosis	Condition	SNOMED	NO	NO	NO
133228	Dental caries	Condition	SNOMED	NO	NO	NO
4207690	Dens in dente	Condition	SNOMED	NO	NO	NO
4270186	Dens evaginatus	Condition	SNOMED	NO	NO	NO

135303	Cyst of oral soft tissue	Condition	SNOMED	NO	NO	NO
4143172	Concrescence of teeth	Condition	SNOMED	NO	NO	NO
138790	Chronic periodontitis	Condition	SNOMED	NO	NO	NO
133794	Chronic gingivitis	Condition	SNOMED	NO	NO	NO
37397422	Asymptomatic periapical periodontitis	Condition	SNOMED	NO	NO	NO
433243	Anomaly of tooth position	Condition	SNOMED	NO	NO	NO
440438	Anodontia	Condition	SNOMED	NO	NO	NO
433229	Ankylosis of tooth	Condition	SNOMED	NO	NO	NO
132943	Acute periodontitis	Condition	SNOMED	NO	NO	NO
138176	Acute gingivitis	Condition	SNOMED	NO	NO	NO
440432	Accretion on teeth	Condition	SNOMED	NO	NO	NO
436445	Abrasion of tooth	Condition	SNOMED	NO	NO	NO
45757201	Abnormal size of tooth	Condition	SNOMED	NO	NO	NO
433514	Abnormal hard tissue formation in pulp	Condition	SNOMED	NO	NO	NO

■ Other ill-defined heart disease

Concept Id	Concept Name	Domain	Vocabulary	Excluded	Descendants	Mapped
432937	Rupture of papillary muscle	Condition	SNOMED	NO	NO	NO
438171	Rupture of chordae tendineae	Condition	SNOMED	NO	NO	NO
321320	Myocardial degeneration	Condition	SNOMED	NO	NO	NO
314658	Cardiomegaly	Condition	SNOMED	NO	NO	NO
4029831	Bacterial infectious disease of heart	Condition	SNOMED	NO	NO	NO
43020480	Acquired coronary artery fistula	Condition	SNOMED	NO	NO	NO
4108950	Acquired cardiac septal defect	Condition	SNOMED	NO	NO	NO

■ Acute kidney injury

Concept Id	Concept Name	Domain	Vocabulary	Excluded	Descendants	Mapped
192359	Renal failure syndrome	Condition	SNOMED	NO	NO	NO
4126305	Acute renal impairment	Condition	SNOMED	NO	NO	NO
197320	Acute renal failure syndrome	Condition	SNOMED	NO	NO	NO

■ Thrombophlebitis/ Thromboembolism

Concept Id	Concept Name	Domain	Vocabulary	Excluded	Descendants	Mapped
4145867	Venous occlusion	Condition	SNOMED	NO	NO	NO
4108680	Thrombosis of atrium, auricular appendage, and ventricle due to and following acute myocardial infarction	Condition	SNOMED	NO	NO	NO
444097	Thrombophlebitis of lower extremities	Condition	SNOMED	NO	NO	NO
439838	Thrombophlebitis migrans	Condition	SNOMED	NO	NO	NO
320741	Thrombophlebitis	Condition	SNOMED	NO	NO	NO
440417	Pulmonary embolism	Condition	SNOMED	NO	NO	NO
199837	Portal vein thrombosis	Condition	SNOMED	NO	NO	NO
440738	Phlebitis of the femoral vein	Condition	SNOMED	NO	NO	NO
438452	Phlebitis of superficial veins of lower extremity	Condition	SNOMED	NO	NO	NO
4237062	Mural thrombus of heart	Condition	SNOMED	NO	NO	NO
435565	Embolism and thrombosis of the vena cava	Condition	SNOMED	NO	NO	NO
193512	Embolism and thrombosis of the renal vein	Condition	SNOMED	NO	NO	NO
198446	Embolism and thrombosis of the abdominal aorta	Condition	SNOMED	NO	NO	NO
314965	Embolism and thrombosis of an arm or leg artery	Condition	SNOMED	NO	NO	NO
4234997	Disorder of vein	Condition	SNOMED	NO	NO	NO
77310	Deep vein phlebitis and thrombophlebitis of the leg	Condition	SNOMED	NO	NO	NO
196715	Budd-Chiari syndrome	Condition	SNOMED	NO	NO	NO
312337	Arterial embolus and thrombosis	Condition	SNOMED	NO	NO	NO
4170620	Aortic thromboembolism	Condition	SNOMED	NO	NO	NO
312927	Acute cor pulmonale	Condition	SNOMED	NO	NO	NO

■ Pneumonia

Concept Id	Concept Name	Domain	Vocabulary	Excluded	Descendants	Mapped
261326	Viral pneumonia	Condition	SNOMED	NO	NO	NO
259852	Staphylococcal pneumonia	Condition	SNOMED	NO	NO	NO
261324	Pneumonia due to Streptococcus	Condition	SNOMED	NO	NO	NO
436145	Pneumonia due to respiratory syncytial virus	Condition	SNOMED	NO	NO	NO
252351	Pneumonia due to Pseudomonas	Condition	SNOMED	NO	NO	NO
253790	Pneumonia due to Klebsiella pneumoniae	Conditi	SNOME	NO	NO	NO

		Conditi on	SNOME D			
258180	Pneumonia due to Gram negative bacteria	Conditi on	SNOME D	NO	NO	NO
260430	Pneumonia due to Escherichia coli	Conditi on	SNOME D	NO	NO	NO
255848	Pneumonia	Conditi on	SNOME D	NO	NO	NO
258785	Pneumococcal pneumonia	Conditi on	SNOME D	NO	NO	NO
440431	Mycoplasma pneumonia	Conditi on	SNOME D	NO	NO	NO
4133224	Lobar pneumonia	Conditi on	SNOME D	NO	NO	NO
443410	Infective pneumonia	Conditi on	SNOME D	NO	NO	NO
260754	Haemophilus influenzae pneumonia	Conditi on	SNOME D	NO	NO	NO
252655	Group B streptococcal pneumonia	Conditi on	SNOME D	NO	NO	NO
4174308	Congenital viral pneumonia	Conditi on	SNOME D	NO	NO	NO
4071610	Congenital staphylococcal pneumonia	Conditi on	SNOME D	NO	NO	NO
4048149	Congenital pseudomonal pneumonia	Conditi on	SNOME D	NO	NO	NO
255084	Congenital pneumonia	Conditi on	SNOME D	NO	NO	NO
4071611	Congenital group B hemolytic streptococcal pneumonia	Conditi on	SNOME D	NO	NO	NO
4048148	Congenital Escherichia coli pneumonia	Conditi on	SNOME D	NO	NO	NO
256722	Bronchopneumonia	Conditi on	SNOME D	NO	NO	NO
257315	Bacterial pneumonia	Conditi on	SNOME D	NO	NO	NO
254677	Adenoviral pneumonia	Conditi on	SNOME D	NO	NO	NO
4025165	Abscess of lung with pneumonia	Conditi on	SNOME D	NO	NO	NO
4025164	Abscess of lung and mediastinum	Conditi on	SNOME D	NO	NO	NO

■ Bronchiolitis/bronchitis

Concept Id	Concept Name	Domain	Vocabulary	Excluded	Descendants	Mapped
256451	Bronchitis	Condition	SNOMED	NO	NO	NO
4052544	Acute viral bronchiolitis	Condition	SNOMED	NO	NO	NO
260139	Acute bronchitis	Condition	SNOMED	NO	NO	NO
260125	Acute bronchiolitis	Condition	SNOMED	NO	NO	NO

■ Obesity

Concept Id	Concept Name	Domain	Vocabula ry	Exclud ed	Descenda nts	Mappe d
4217557	Simple obesity	Conditi	SNOME	NO	NO	NO

		on	D			
433736	Obesity	Condition	SNOMED	NO	NO	NO
4100857	Extreme obesity with alveolar hypoventilation	Condition	SNOMED	NO	NO	NO

■ Communication/motor symptoms

Concept Id	Concept Name	Domain	Vocabulary	Excluded	Descendants	Mapped
436667	Stuttering	Condition	SNOMED	NO	NO	NO
443443	Receptive language disorder	Condition	SNOMED	NO	NO	NO
4039744	Phonological disorder	Condition	SNOMED	NO	NO	NO
4047124	Expressive language disorder	Condition	SNOMED	NO	NO	NO
435232	Disorder of speech and language development	Condition	SNOMED	NO	NO	NO
436373	Developmental speech disorder	Condition	SNOMED	NO	NO	NO
4046219	Acquired epileptic aphasia	Condition	SNOMED	NO	NO	NO

■ Gastroenteritis

Concept Id	Concept Name	Domain	Vocabulary	Excluded	Descendants	Mapped
196895	Viral gastroenteritis due to Norwalk-like agent	Condition	SNOMED	NO	NO	NO
196620	Viral enteritis	Condition	SNOMED	NO	NO	NO
198678	Intestinal infectious disease	Condition	SNOMED	NO	NO	NO
4043371	Inflammatory disorder of digestive tract	Condition	SNOMED	NO	NO	NO
4341633	Indeterminate colitis	Condition	SNOMED	NO	NO	NO
201618	Disorder of intestine	Condition	SNOMED	NO	NO	NO
4297887	Disorder of digestive organ	Condition	SNOMED	NO	NO	NO

■ Arrhythmias

Concept Id	Concept Name	Domain	Vocabulary	Excluded	Descendants	Mapped
4103295	Ventricular tachycardia	Condition	SNOMED	NO	NO	NO
4089462	Ventricular premature complex	Condition	SNOMED	NO	NO	NO
4185572	Ventricular arrhythmia	Condition	SNOMED	NO	NO	NO
36714994	Typical atrial flutter	Condition	SNOMED	NO	NO	NO

321315	Trifascicular block	Condition	SNOMED	NO	NO	NO
4275423	Supraventricular tachycardia	Condition	SNOMED	NO	NO	NO
4317150	Sudden cardiac death	Condition	SNOMED	NO	NO	NO
4261842	Sick sinus syndrome	Condition	SNOMED	NO	NO	NO
318448	Second degree atrioventricular block	Condition	SNOMED	NO	NO	NO
314059	Right bundle branch block	Condition	SNOMED	NO	NO	NO
4111552	Re-entry ventricular arrhythmia	Condition	SNOMED	NO	NO	NO
4232697	Persistent atrial fibrillation	Condition	SNOMED	NO	NO	NO
313792	Paroxysmal tachycardia	Condition	SNOMED	NO	NO	NO
4154290	Paroxysmal atrial fibrillation	Condition	SNOMED	NO	NO	NO
4268046	Left posterior fascicular block	Condition	SNOMED	NO	NO	NO
316998	Left bundle branch block	Condition	SNOMED	NO	NO	NO
4295336	Left anterior fascicular block	Condition	SNOMED	NO	NO	NO
4088351	Junctional premature complex	Condition	SNOMED	NO	NO	NO
4166844	Intraventricular conduction defect	Condition	SNOMED	NO	NO	NO
320425	Heart block	Condition	SNOMED	NO	NO	NO
314379	First degree atrioventricular block	Condition	SNOMED	NO	NO	NO
316999	Conduction disorder of the heart	Condition	SNOMED	NO	NO	NO
320744	Complete atrioventricular block	Condition	SNOMED	NO	NO	NO
4141360	Chronic atrial fibrillation	Condition	SNOMED	NO	NO	NO
45766277	Cardiac implant in situ	Condition	SNOMED	NO	NO	NO
44784217	Cardiac arrhythmia	Condition	SNOMED	NO	NO	NO
4120088	Cardiac arrest with successful resuscitation	Condition	SNOMED	NO	NO	NO
321042	Cardiac arrest	Condition	SNOMED	NO	NO	NO
313791	Bundle branch block	Condition	SNOMED	NO	NO	NO
4250169	Bifascicular block	Condition	SNOMED	NO	NO	NO
36712986	Atypical atrial flutter	Condition	SNOMED	NO	NO	NO
316135	Atrioventricular block	Condition	SNOMED	NO	NO	NO
4115173	Atrial premature complex	Condition	SNOMED	NO	NO	NO
4068155	Atrial arrhythmia	Condition	SNOMED	NO	NO	NO

4057008	Accelerated atrioventricular conduction	Condition	SNOMED	NO	NO	NO
4091901	Aberrant premature complexes	Condition	SNOMED	NO	NO	NO

■ Dysautonomia

Concept Id	Concept Name	Domain	Vocabulary	Excluded	Descendants	Mapped
4159659	Postural orthostatic tachycardia syndrome	Condition	SNOMED	NO	NO	NO
194913	Idiopathic peripheral autonomic neuropathy	Condition	SNOMED	NO	NO	NO
434633	Disorder of autonomic nervous system	Condition	SNOMED	NO	NO	NO

■ Depression

Concept Id	Concept Name	Domain	Vocabulary	Excluded	Descendants	Mapped
435220	Severe recurrent major depression without psychotic features	Condition	SNOMED	NO	NO	NO
4327337	Severe major depression without psychotic features	Condition	SNOMED	NO	NO	NO
4250023	Severe major depression with psychotic features	Condition	SNOMED	NO	NO	NO
434911	Recurrent major depressive episodes, severe, with psychosis	Condition	SNOMED	NO	NO	NO
433991	Recurrent major depression in remission	Condition	SNOMED	NO	NO	NO
4282316	Recurrent major depression	Condition	SNOMED	NO	NO	NO
4098302	Recurrent depression	Condition	SNOMED	NO	NO	NO
373176	Organic mood disorder	Condition	SNOMED	NO	NO	NO
444100	Mood disorder	Condition	SNOMED	NO	NO	NO
4077577	Moderate recurrent major depression	Condition	SNOMED	NO	NO	NO
4307111	Moderate major depression	Condition	SNOMED	NO	NO	NO
4228802	Mild recurrent major depression	Condition	SNOMED	NO	NO	NO
4336957	Mild major depression	Condition	SNOMED	NO	NO	NO
4060424	Mental disorders during pregnancy, childbirth and the puerperium	Condition	SNOMED	NO	NO	NO
442919	Mental disorder in the puerperium - baby delivered	Condition	SNOMED	NO	NO	NO
4169106	Feeling unhappy	Condition	SNOMED	NO	NO	NO
433440	Dysthymia	Condition	SNOMED	NO	NO	NO
440383	Depressive disorder	Condition	SNOMED	NO	NO	NO

440696	Cyclothymia	Condit ion	SNOM ED	NO	NO	NO

■ Pericarditis

Concept Id	Concept Name	Domain	Vocabular y	Exclude d	Descendan ts	Mappe d
4138837	Pericarditis	Conditio n	SNOME D	NO	NO	NO
4108814	Pericardial effusion - noninflammatory	Conditio n	SNOME D	NO	NO	NO
4217075	Infectious pericarditis	Conditio n	SNOME D	NO	NO	NO
258449	Hemopericardium	Conditio n	SNOME D	NO	NO	NO
318772	Disorder of pericardium	Conditio n	SNOME D	NO	NO	NO
321307	Chronic rheumatic pericarditis	Conditio n	SNOME D	NO	NO	NO
4203625	Chronic constrictive pericarditis	Conditio n	SNOME D	NO	NO	NO
4181182	Chronic adhesive pericarditis	Conditio n	SNOME D	NO	NO	NO
321586	Acute rheumatic pericarditis	Conditio n	SNOME D	NO	NO	NO
320116	Acute pericarditis	Conditio n	SNOME D	NO	NO	NO
315293	Acute idiopathic pericarditis	Conditio n	SNOME D	NO	NO	NO

■ Psychotic disorder

Conce pt Id	Concept Name	Doma in	Vocab ulary	Exclu ded	Descen dants	Map ped
43351 68	Persistent delusional disorder	Condi tion	SNO MED	NO	NO	NO
41002 47	Organic psychotic condition	Condi tion	SNO MED	NO	NO	NO
37317 5	Organic hallucinosis	Condi tion	SNO MED	NO	NO	NO
41011 49	Non-organic psychosis	Condi tion	SNO MED	NO	NO	NO
43258 6	Mental disorder	Condi tion	SNO MED	NO	NO	NO
43695 2	Induced psychotic disorder	Condi tion	SNO MED	NO	NO	NO
43259 0	Delusional disorder	Condi tion	SNO MED	NO	NO	NO
43351 69	Acute transient psychotic disorder	Condi tion	SNO MED	NO	NO	NO
41334 95	Acute schizophrenia-like psychotic disorder	Condi tion	SNO MED	NO	NO	NO
37016 719	Acute polymorphic psychotic disorder without symptoms of schizophrenia	Condi tion	SNO MED	NO	NO	NO
37016	Acute polymorphic psychotic disorder co-occurrent	Condi	SNO	NO	NO	NO

741	with symptoms of schizophrenia	tion	MED			

■ Pulmonary fibrosis

Concept Id	Concept Name	Domain	Vocabulary	Excluded	Descendants	Mapped
4197819	Fibrosis of lung	Condition	SNOMED	NO	NO	NO

■ Seizure/epilepsy

Concept Id	Concept Name	Domain	Vocabulary	Excluded	Descendants	Mapped
765514	Tonic-clonic seizure, refractory	Condition	SNOMED	NO	NO	NO
762957	Tonic-clonic seizure, non-refractory	Condition	SNOMED	NO	NO	NO
4183856	Tonic-clonic seizure	Condition	SNOMED	NO	NO	NO
43530626	Simple partial seizure	Condition	SNOMED	NO	NO	NO
4196708	Seizure related finding	Condition	SNOMED	NO	NO	NO
4029498	Seizure disorder	Condition	SNOMED	NO	NO	NO
377091	Seizure	Condition	SNOMED	NO	NO	NO
4029783	Partial seizure with impaired consciousness	Condition	SNOMED	NO	NO	NO
4106574	Partial seizure	Condition	SNOMED	NO	NO	NO
4101747	Localization-related(focal)(partial)idiopathic epilepsy and epileptic syndromes with seizures of localized onset	Condition	SNOMED	NO	NO	NO
4274575	Idiopathic generalized epilepsy	Condition	SNOMED	NO	NO	NO
4263736	Generalized-onset seizures	Condition	SNOMED	NO	NO	NO
4078333	Generalized seizure	Condition	SNOMED	NO	NO	NO
4055361	Generalized epilepsy	Conditio	SNOMED	NO	NO	NO

		n				
4047897	Epilepsy with grand mal seizures on awakening	Condition	SNOMED	NO	NO	NO
40483585	Epilepsy characterized by intractable complex partial seizures	Condition	SNOMED	NO	NO	NO
380378	Epilepsy	Condition	SNOMED	NO	NO	NO
374023	Epilepsia partialis continua	Condition	SNOMED	NO	NO	NO
4236312	Complex partial epileptic seizure	Condition	SNOMED	NO	NO	NO
4179936	Childhood absence epilepsy	Condition	SNOMED	NO	NO	NO
4194232	Absence seizure	Condition	SNOMED	NO	NO	NO

■ Lupus

Concept Id	Concept Name	Domain	Vocabulary	Excluded	Descendants	Mapped
4063582	Systemic sclerosis induced by drugs and chemicals	Condition	SNOMED	NO	NO	NO
134442	Systemic sclerosis	Condition	SNOMED	NO	NO	NO
4344158	Systemic lupus erythematosus with organ/system involvement	Condition	SNOMED	NO	NO	NO
257628	Systemic lupus erythematosus	Condition	SNOMED	NO	NO	NO
4343923	Subacute cutaneous lupus erythematosus	Condition	SNOMED	NO	NO	NO
254443	Sjögren's syndrome	Condition	SNOMED	NO	NO	NO
4141144	Relapsing febrile nodular nonsuppurative panniculitis	Condition	SNOMED	NO	NO	NO
40485046	Progressive systemic sclerosis	Condition	SNOMED	NO	NO	NO
80800	Polymyositis	Condition	SNOMED	NO	NO	NO
255348	Polymyalgia rheumatica	Condition	SNOMED	NO	NO	NO
4079978	Overlap syndrome	Condition	SNOMED	NO	NO	NO
4009035	Multifocal fibrosclerosis	Condition	SNOMED	NO	NO	NO
255891	Lupus erythematosus	Condition	SNOMED	NO	NO	NO
76790	Hypermobility syndrome	Condition	SNOMED	NO	NO	NO
4083100	Fasciitis with eosinophilia syndrome	Condition	SNOMED	NO	NO	NO
4063581	Drug-induced systemic lupus erythematosus	Condition	SNOMED	NO	NO	NO

4066824	Discoid lupus erythematosus	Condition	SNOMED	NO	NO	NO
4344161	Dermatomyositis with malignant disease	Condition	SNOMED	NO	NO	NO
80182	Dermatomyositis	Condition	SNOMED	NO	NO	NO
4135937	CREST syndrome	Condition	SNOMED	NO	NO	NO
4005037	Childhood type dermatomyositis	Condition	SNOMED	NO	NO	NO
436642	Behcet's syndrome	Condition	SNOMED	NO	NO	NO

■ Thyroiditis

Concept Id	Concept Name	Domain	Vocabulary	Excluded	Descendants	Mapped
140976	Toxic uninodular goiter	Condition	SNOMED	NO	NO	NO
135778	Toxic multinodular goiter	Condition	SNOMED	NO	NO	NO
138717	Toxic diffuse goiter	Condition	SNOMED	NO	NO	NO
4177975	Thyrotoxicosis factitia	Condition	SNOMED	NO	NO	NO
138387	Thyrotoxicosis	Condition	SNOMED	NO	NO	NO
133436	Thyrotoxic crisis	Condition	SNOMED	NO	NO	NO
133444	Thyroiditis	Condition	SNOMED	NO	NO	NO
132579	Subacute thyroiditis	Condition	SNOMED	NO	NO	NO
4337834	Hyperthyroidism due to ectopic thyroid nodule	Condition	SNOMED	NO	NO	NO
4142479	Hyperthyroidism	Condition	SNOMED	NO	NO	NO
4130020	Drug-induced thyroiditis	Condition	SNOMED	NO	NO	NO
4129364	Chronic thyroiditis with transient thyrotoxicosis	Condition	SNOMED	NO	NO	NO
137520	Chronic thyroiditis	Condition	SNOMED	NO	NO	NO
4281109	Autoimmune thyroiditis	Condition	SNOMED	NO	NO	NO
133737	Acute thyroiditis	Condition	SNOMED	NO	NO	NO

■ Septicemia

Concept Id	Concept Name	Domain	Vocabulary	Excluded	Descendants	Mapped
442137	Septicemic plague	Condition	SNOMED	NO	NO	NO
4048594	Sepsis of newborn due to	Conditi	SNOME	NO	NO	NO

	Staphylococcus aureus	on	D			
46270041	Sepsis of newborn due to group B Streptococcus	Condition	SNOMED	NO	NO	NO
4071727	Sepsis of newborn due to Escherichia coli	Condition	SNOMED	NO	NO	NO
4048275	Sepsis of newborn due to anaerobes	Condition	SNOMED	NO	NO	NO
40487616	Sepsis due to Streptococcus pyogenes	Condition	SNOMED	NO	NO	NO
40489912	Sepsis due to Streptococcus pneumoniae	Condition	SNOMED	NO	NO	NO
40489910	Sepsis due to Streptococcus group D	Condition	SNOMED	NO	NO	NO
40489909	Sepsis due to Streptococcus agalactiae	Condition	SNOMED	NO	NO	NO
40489907	Sepsis due to Staphylococcus aureus	Condition	SNOMED	NO	NO	NO
40487059	Sepsis due to Staphylococcus	Condition	SNOMED	NO	NO	NO
40493039	Sepsis due to Salmonella	Condition	SNOMED	NO	NO	NO
40486685	Sepsis due to Listeria monocytogenes	Condition	SNOMED	NO	NO	NO
40486059	Sepsis due to Haemophilus influenzae	Condition	SNOMED	NO	NO	NO
40493038	Sepsis due to Gram negative bacteria	Condition	SNOMED	NO	NO	NO
40486058	Sepsis due to Erysipelothrix	Condition	SNOMED	NO	NO	NO
40486629	Sepsis due to Candida	Condition	SNOMED	NO	NO	NO
40489979	Sepsis due to Bacillus anthracis	Condition	SNOMED	NO	NO	NO
40486631	Sepsis due to anaerobic bacteria	Condition	SNOMED	NO	NO	NO
40487063	Sepsis due to Actinomyces	Condition	SNOMED	NO	NO	NO
132797	Sepsis	Condition	SNOMED	NO	NO	NO
4102318	Puerperal sepsis	Condition	SNOMED	NO	NO	NO
133691	Meningococcemia	Condition	SNOMED	NO	NO	NO
4029319	Disseminated herpes simplex	Condition	SNOMED	NO	NO	NO
4345708	Chronic meningococcemia	Condition	SNOMED	NO	NO	NO
133594	Bacterial sepsis of newborn	Condition	SNOMED	NO	NO	NO
4090689	Acute meningococcemia	Condition	SNOMED	NO	NO	NO

■ Tonsillitis

Concept Id	Concept Name	Domain	Vocabulary	Excluded	Descendants	Mapped

4148121	Tonsil and/or adenoid hypertrophy	Condition	SNOMED	NO	NO	NO
4212727	Streptococcal tonsillitis	Condition	SNOMED	NO	NO	NO
4110362	Recurrent acute tonsillitis	Condition	SNOMED	NO	NO	NO
42534820	Recurrent acute streptococcal tonsillitis	Condition	SNOMED	NO	NO	NO
440751	Peritonsillar abscess	Condition	SNOMED	NO	NO	NO
28457	Hypertrophy of tonsils	Condition	SNOMED	NO	NO	NO
438788	Hypertrophy of adenoids	Condition	SNOMED	NO	NO	NO
23220	Chronic tonsillitis	Condition	SNOMED	NO	NO	NO
432347	Chronic disease of tonsils AND/OR adenoids	Condition	SNOMED	NO	NO	NO
24660	Acute tonsillitis	Condition	SNOMED	NO	NO	NO

■ Coagulation/hemorrhagic disorders

Concept Id	Concept Name	Domain	Vocabulary	Excluded	Descendants	Mapped
434316	von Willebrand disorder	Condition	SNOMED	NO	NO	NO
4125650	Thrombophilia	Condition	SNOMED	NO	NO	NO
432870	Thrombocytopenic disorder	Condition	SNOMED	NO	NO	NO
40321716	Secondary thrombocytopenia	Condition	SNOMED	NO	NO	NO
437241	Qualitative platelet disorder	Condition	SNOMED	NO	NO	NO
441264	Primary thrombocytopenia	Condition	SNOMED	NO	NO	NO
441259	Non-thrombocytopenic purpura	Condition	SNOMED	NO	NO	NO
4160048	Multiple bruising	Condition	SNOMED	NO	NO	NO
4137430	Idiopathic thrombocytopenic purpura	Condition	SNOMED	NO	NO	NO
4231770	Hereditary thrombophilia	Condition	SNOMED	NO	NO	NO
437256	Hereditary factor XI deficiency disease	Condition	SNOMED	NO	NO	NO
434007	Hereditary factor VIII deficiency disease	Condition	SNOMED	NO	NO	NO
436678	Hereditary factor IX deficiency disease	Condition	SNOMED	NO	NO	NO
4101602	Henoch-Schönlein purpura	Condition	SNOMED	NO	NO	NO
432869	Hemorrhagic disorder due to circulating anticoagulants	Condition	SNOMED	NO	NO	NO
4214869	Hemophilic arthropathy	Condition	SNOMED	NO	NO	NO

4314452	Easy bruising	Condition	SNOMED	NO	NO	NO
436093	Disseminated intravascular coagulation	Condition	SNOMED	NO	NO	NO
4179872	Disorder of hemostatic system	Condition	SNOMED	NO	NO	NO
432296	Coagulation factor deficiency syndrome	Condition	SNOMED	NO	NO	NO
4058577	Bruising symptom	Condition	SNOMED	NO	NO	NO
432585	Blood coagulation disorder	Condition	SNOMED	NO	NO	NO
437312	Bleeding	Condition	SNOMED	NO	NO	NO
432863	Acquired coagulation factor deficiency	Condition	SNOMED	NO	NO	NO

■ Urinary tract infection

Concept Id	Concept Name	Domain	Vocabulary	Excluded	Descendants	Mapped
81902	Urinary tract infectious disease	Condition	SNOMED	NO	NO	NO
4024000	Urinary system finding	Condition	SNOMED	NO	NO	NO
195862	Urethritis	Condition	SNOMED	NO	NO	NO
196733	Urethral syndrome	Condition	SNOMED	NO	NO	NO
195313	Urethral abscess	Condition	SNOMED	NO	NO	NO
4177206	Tubulointerstitial nephritis	Condition	SNOMED	NO	NO	NO
441615	Trigonitis	Condition	SNOMED	NO	NO	NO
198192	Renal and perinephric abscess	Condition	SNOMED	NO	NO	NO
4174994	Pyonephrosis	Condition	SNOMED	NO	NO	NO
201792	Nongonococcal urethritis	Condition	SNOMED	NO	NO	NO
194685	Non-obstructive reflux-associated chronic pyelonephritis	Condition	SNOMED	NO	NO	NO
197684	Dysuria	Condition	SNOMED	NO	NO	NO
200450	Disorder of urethra	Condition	SNOMED	NO	NO	NO
75865	Disorder of the urinary system	Condition	SNOMED	NO	NO	NO
4264718	Chronic tubulointerstitial nephritis	Condition	SNOMED	NO	NO	NO
4126297	Chronic obstructive pyelonephritis	Condition	SNOMED	NO	NO	NO
75863	Chronic interstitial cystitis	Condition	SNOMED	NO	NO	NO
201621	Chronic cystitis	Condition	SNOMED	NO	NO	NO

4102631	Acute tubulointerstitial nephritis	Condition	SNOMED	NO	NO	NO
194081	Acute cystitis	Condition	SNOMED	NO	NO	NO
